# Charting the Scientific Landscape of Indirect Estimation Models in Doping Prevalence Research: A Bibliometric Analysis with Narrative Appraisal

**DOI:** 10.3390/sports14060229

**Published:** 2026-06-03

**Authors:** Andrea Petróczi, Dominic Sagoe, Anna Kiss, Sándor Soós, Razieh Chegeni, Annalena Veltmaat, Maarten Cruyff, Peter van der Heijden, Olivier de Hon

**Affiliations:** 1 Faculty of Health and Sport Sciences, Széchenyi István University, 9026 Győr, Hungary; petroczi.andrea@sze.hu; 2 Department of Psychosocial Science, University of Bergen, 5020 Bergen, Norway; 3 Human Enhancement and Body Image Lab (HEBI Lab), Addiction Research Group, University of Bergen, 5015 Bergen, Norway; razieh.chegeni@psykologi.uio.no; 4 Department of Science Policy and Scientometrics, Hungarian Academy of Sciences (MTA), 1051 Budapest, Hungary; kiss.anna891@gmail.com (A.K.); soossand@gmail.com (S.S.); 5 Faculty of Education and Psychology, ELTE Eötvös Loránd University, 1075 Budapest, Hungary; 6 PROMENTA Research Center, Department of Psychology, University of Oslo, 0317 Oslo, Norway; 7 Department of Sport and Sports Science, TU Dortmund University, 44227 Dortmund, Germany; annalena.veltmaat@tu-dortmund.de; 8 Faculty of Social Sciences, Utrecht University, 3508 TC Utrecht, The Netherlands; m.cruyff@uu.nl (M.C.); p.g.m.vanderheijden@uu.nl (P.v.d.H.); 9 Southampton Statistical Sciences Research Institute, University of Southampton, Southampton SO17 1BJ, UK; 10 Doping Authority Netherlands, 2900 EA Capelle aan den IJssel, The Netherlands; o.dehon@dopingautoriteit.nl

**Keywords:** doping prevalence, sport, survey, indirect estimation, randomized response, narrative review, bibliometric mapping

## Abstract

Interpreting doping prevalence estimates generated through indirect estimation models (IEMs) remains challenging for sport policy and governance due to the wide variation in reported rates and methodological complexity. In this study, we combined a critical appraisal of the methodological and epistemic developments of IEM applications to doping prevalence with a bibliometric analysis of publication trends, citation patterns, and collaboration networks, using a convergent parallel mixed-methods design. Across 52 records published between 2002 and 2026, this study maps the scientific landscape of IEM-based doping prevalence research. Findings show that IEM-based prevalence research is methodologically sophisticated yet institutionally dispersed and largely Eurocentric, reflecting a field still consolidating its standards and disciplinary identity. Over time, the focus has shifted from reporting prevalence rates to methodological critique and re-analysis of existing datasets. Reported prevalence estimates, ranging from 0 to 57.1%, are highly sensitive to modelling assumptions about athlete behaviour in complex survey environments. While this trend strengthens rigour, it also complicates evidence synthesis for policy actors and risks undermining trust in IEM-based estimates if poorly communicated. Anti-doping organisations and researchers should treat IEM-derived prevalence as bounded indicators rather than definitive rates and integrate prevalence evidence with contextual data for transparent policy and public communication.

## 1. Introduction

High prevalence estimates are routinely mobilised as rhetorical justification for new studies and policy arguments stating that doping is widespread, underdetected, and/or presents an urgent need in sport [1,2,3,4,5]. Yet, despite decades of surveillance and testing, a persistent gap remains between what anti-doping systems can detect and what athletes may actually do, resulting in one of the most enduring gaps in sport governance [6]. Biological testing detects incidences, not prevalence, and conventional self-reports are vulnerable to denial, fear of exposure, and social desirability bias [7,8]. In a domain where concealment is structurally embedded, empirical observation is necessarily limited [9,10,11,12,13]. Factors mitigating against socially desirable responding include survey mode (e.g., online administration, full anonymity), proxy reporting (i.e., projected responding for someone else, such as ‘an elite cyclist’ or ‘an American athlete’), face-saving strategies (e.g., using forgiving words or adding a forgiving preamble), the deceptive bogus pipeline, implicit associations, and indirect estimate models (IEMs) using list experiments or probability-based techniques [14,15].

Although it is intuitively appealing to assume that asking athletes directly should provide insight into prohibited practices, eliciting honest self-admission of doping is neither simple nor straightforward. The potential consequences of disclosure [16,17], combined with the strong social stigma attached to doping [18], mean that even surveys conducted under assured anonymity cannot be assumed to yield truthful responses. Behind every prevalence statistic lies both a practical and an ethical dilemma: how to measure behaviours that individuals are strongly motivated to conceal. To address this challenge, researchers studying doping prevalence (and other socially sensitive or transgressive behaviours) have increasingly turned to indirect survey approaches designed to reduce response bias due to fear of exposure, and thus enable more honest reporting.

### 1.1. Estimating the Prevalence of Sensitive Behaviour with Indirect Estimation Models

Indirect estimation models (IEMs) comprise a family of innovative survey techniques designed to protect both respondents and researchers by creating safe survey conditions that go beyond conventional anonymity [19,20,21]. By design, IEMs obscure the link between an individual’s response and the sensitive behaviour being assessed. Even when a respondent’s answer to a survey item is known, it is impossible to determine whether it represents an admission or denial of doping because response options are intentionally masked via randomising or unrelated mechanisms. This feature provides a crucial procedural and psychological safeguard for respondents and ensures that individual identification—and by extension, sanction or prosecution—is impossible.

IEMs achieve this protection through different masking mechanisms. Some variants, including forced response (FR) (proposed by Boruch [22]), Kuk’s model [23], and the Unrelated Question Model (UQM) [24,25], obscure which question—sensitive or unrelated/innocuous—is being answered. In these designs, only a random subsample receives the sensitive question, while others respond to an unrelated item with a known probability distribution (Appendix A Table A1). In other models, such as the Crosswise Model (CM) [26] and the Single Sample Count (SSC) [27,28], respondents are never required to directly admit the undesirable behaviour. Instead, their answer to the sensitive item is combined with an unrelated question, producing a composite response that conceals whether the respondent has engaged in prohibited practices. Across these approaches, only the respondent knows which question is being answered, not the researcher. Together, these mechanisms make IEMs uniquely suited for collecting data on socially sensitive or prohibited behaviours such as doping while maintaining the ethical integrity of both participants and investigators [20,21].

Survey instruction noncompliance, arising for various reasons, is a key vulnerability of IEMs because it biases prevalence estimates. While noncompliance affects all self-report methods, it is particularly salient in IEMs due to their instructional complexity, which may elevate noncompliance rates [29,30,31,32]. At the same time, IEMs uniquely allow the magnitude of noncompliance to be estimated and potentially corrected for, which is an option mostly unavailable in direct self-reports. Analytical approaches to identifying and adjusting for survey instruction noncompliance are, in principle, applicable across IEMs. In practice, however, most models require empirical manipulation at the data collection stage, typically via two parallel survey versions or additional experimental conditions, to formally test noncompliance and enable retrospective statistical adjustments. Without such design features, noncompliance remains largely unobservable and is implicitly absorbed into prevalence estimates. One practical exception is SSC, which, unlike other models summarised in Table 1, does not require parallel sampling frames or additional experimental conditions. Its structure allows post hoc assessment of response irregularities using a single dataset, reducing logistical burden, though with interpretive trade-offs and wider confidence intervals [28].

#### 1.1.1. Behavioural Aspects and Interpretive Risk

From a regulatory perspective, differences between IEMs extend beyond statistical properties to how surveys are experienced by respondents. A key consideration is face validity, understood here as whether a survey clearly appears to be about doping prevalence and whether respondents feel their participation meaningfully contributes to that aim. Models with high face validity ensure that all respondents perceive themselves as answering the doping question, which may support engagement and compliance, but can also increase perceived personal risk if protection mechanisms are not well understood. Forced-response variants present a distinct trade-off: by design, some respondents must give an affirmative (“yes”) response irrespective of their true behaviour. This enhances anonymity because the researcher cannot distinguish between genuine and forced admissions; however, being instructed to say “yes” to a normatively charged question like doping may feel uncomfortable or ethically troubling for some participants [29]. Such discomfort can manifest as partial noncompliance, refusal, or evasive responding, potentially affecting data quality in ways not always visible in final prevalence estimates.

Across IEMs, several regulatory implications apply regardless of design. Prevalence estimates can support situational awareness (e.g., whether doping is plausibly present beyond negligible levels or whether it changes within the same population over time), but they cannot support absolute prevalence thresholds, individual attribution, or direct enforcement logic. Differences between estimates should not be interpreted straightforwardly as differences in underlying behaviour, as they may reflect variation in protection, respondent comfort, or model assumptions. The main regulatory risk therefore lies less in the use of IEMs per se than in misinterpretation—when prevalence figures are detached from their methodological and experiential context, higher estimates may be read as unequivocal indicators of regulatory failure, while lower or revised estimates may be treated with suspicion or attributed to political motives. Understanding how face validity, forced responses, and respondent experience shape estimates is essential to prevent overconfidence, selective citation, and policy decisions driven more by numerical visibility than evidential nuance.

#### 1.1.2. Protecting Both Sides: Respondent and Researcher

An often-overlooked implication is that IEM-based methods also protect the researcher. In traditional self-report surveys, collecting identifiable data on prohibited behaviour could, in theory, expose investigators to knowledge of individual admissions. For investigators who are also practitioners bound by the World Anti-Doping Agency (WADA) Code, such knowledge could create an ethical dilemma due to conflicting duties: confidentiality toward respondents versus obligations to report known dopers to regulatory bodies. Because IEMs prevent anyone (including the researchers) from knowing which respondents have admitted to doping, they remove this potential conflict of duty reporting transgression. Doping prevalence is estimated only at the aggregate level, without identifying individuals. Thus, IEMs perform a dual protective function by promoting safe, honest disclosure among participants while safeguarding researchers from moral and professional jeopardy. The appeal of IEMs in doping research is therefore evident. When applied to doping, IEMs have consistently revealed prevalence rates far exceeding those indicated by positive test results [6], and this is frequently interpreted as a more realistic view of hidden dimensions of sport [33,34].

#### 1.1.3. Limitations of IEMs and Methodological Refinements

Despite their advantages, IEMs are not without limitations. These models rely on probabilistic assumptions. In FR, the probability of answering the sensitive question is known; in UQM, the probability of answering the unrelated question is predetermined; and in SSC and CM, the probability of an affirmative response to the unrelated question is known. IEMs use this information to estimate the proportion of affirmative responses (admission rate) to the sensitive target question. However, estimates depend on respondent comprehension, compliance, and honesty—conditions that cannot always be assumed. Social desirability bias can still influence responses [35], leading some participants to provide self-protective answers irrespective of safeguards. Moreover, the relative complexity of IEM instructions places cognitive and attentional demands on respondents. Variations in reading comprehension, understanding of randomisation mechanisms, or willingness to follow instructions precisely can result in careless or random responding [36,37]. Respondents may also engage in satisficing by speeding through without full engagement, which can also distort prevalence estimates [38,39,40].

Both intentional and unintentional response errors can introduce substantial bias. In response, methodological innovations have been proposed, including cheating detector variants [41,42,43,44] and parallel-form designs [45,46] that allow post hoc adjustments. Such adjustments are typically based on statistical assumptions about the likely extent and nature of noncompliance [44,47], reflecting ongoing efforts to balance rigour, cognitive feasibility, and respondent trust in research on sensitive behaviours. Other approaches combine IEMs with direct questioning to mitigate against self-protective noncompliance [48,49].

### 1.2. Estimated Doping Prevalence and Its Interpretation

The most comprehensive synthesis of IEM-based doping prevalence research is the systematic review and meta-analysis by Sagoe et al. [50], which reviewed 46 outputs published between 2002 and 2024 and aggregated prevalence estimations from 34 studies, covering more than 43,000 athletes. Across multiple IEMs (including the Unmatched Count Technique, FR, CM, and related designs), pooled lifetime prevalence was estimated at 22.5% among competitive athletes (14.3% past-year) and 17.2% among recreational sport participants (10.3% past-year). However, interpretive uncertainty remains substantial: definitions of doping were inconsistent across studies, and survey instruction noncompliance (averaging nearly 29%) was infrequently measured or reported.

For non-expert audiences, including policymakers and sport administrators, navigating divergent prevalence estimates produced by different indirect models is particularly challenging. Without a clear understanding of how these figures are generated and what they can meaningfully represent, estimates risk being compared across incompatible methods or selectively mobilised to support predetermined narratives. IEM-based prevalence estimates are intrinsically model-dependent and contingent on methodological assumptions, including the functioning of randomisation devices and respondents’ comprehension and compliance with complex instructions. Compared with conventional self-report surveys, IEMs impose higher cognitive demands and thereby introduce additional sources of bias and uncertainty [32,34,40,51]. Despite these limitations, IEM-based estimates are often treated in public and policy discourse as definitive indicators of the scale of doping, stripped of methodological conditions. Media coverage tends to privilege striking figures [52,53,54], while subsequent methodological re-analyses or refinements may be framed as minimising the problem rather than legitimate scientific scrutiny [55].

### 1.3. Research Context and Aims

Systematic reviews and meta-analyses are essential for summarising reported prevalence ranges, but they offer limited insight into the epistemic processes through which prevalence estimates are produced, interpreted, circulated, and rendered authoritative. The present study does not seek to establish or reassess the prevalence of doping. Rather, it examines how prevalence estimates derived from indirect estimation models (IEMs) are generated and interpreted within the scientific literature. Combining bibliometric mapping with content-informed interpretation adds analytical depth by demonstrating that prevalence estimates are not neutral metrics, but products of evolving methodological traditions, collaboration networks, and underlying assumptions. Bibliometric mapping can identify patterns of influence, visibility, and methodological dominance, while interpretive synthesis situates these patterns within broader scientific and policy contexts.

Since the early 2000s, IEMs have been increasingly applied to estimate doping prevalence, resulting in a substantial yet uneven body of research [50]. To elucidate how IEM-based prevalence estimates are constructed, interpreted, and embedded within the field, the present study furthers the research of Sagoe et al. [50] through bibliometric mapping combined with content-informed critical reflection. This integrated approach focuses on the structural, collaborative, and conceptual development of IEM-based prevalence research, with the aim of enhancing methodological transparency and informing more nuanced interpretation of survey-based estimates, rather than determining the true prevalence of doping itself.

## 2. Methods

### 2.1. Study Design

**Figure 1:**
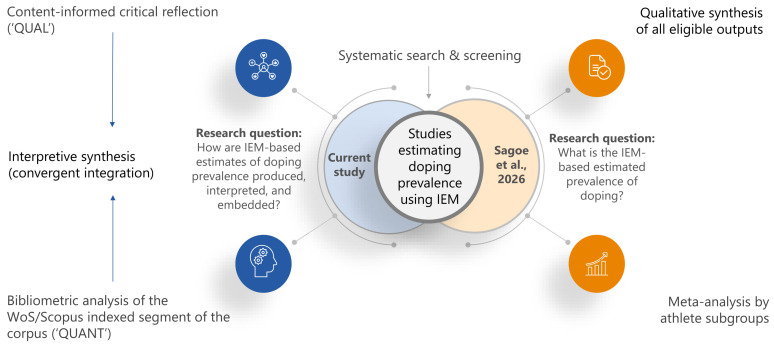
Shared corpus and complementary analytic outputs between Sagoe et al. 2026 [50] and the current study.

Both the current study and the companion systematic review and meta-analysis by Sagoe and colleagues [50] originate from the same systematic search and screening process, pre-registered in PRISMA PROSPERO (CRD42022373691), and, therefore, draw on a common corpus of studies estimating doping prevalence using IEMs (see Figure 1). The companion paper by Sagoe et al. addresses the question “What is the IEM-based estimated prevalence of doping?” and reports a qualitative synthesis of all eligible outputs, alongside a meta-analysis by athlete subgroups [50].

The current study addresses the question “How are IEM-based estimates of doping prevalence produced, interpreted, and embedded?” using a parallel convergent mixed-methods design [56]. As part of this approach, bibliometric mapping of the WoS/Scopus-indexed segment of the corpus (QUANT) and a content-informed critical reflection based on full-text reading (QUAL) are conducted concurrently and then integrated through interpretive synthesis (convergent integration) to generate contextualised implications and directions for future research.

The quantitative (bibliometric) strand maps publication trends, citation impact, and collaboration networks within research on IEM-based doping prevalence estimation. The qualitative strand complements this by examining, at the level of article content, the conceptual evolution of the field, methodological debates, and the theoretical assumptions underpinning the use of IEMs. Integrating these strands provides both structural insights (how the literature is organised and connected) and interpretive insights (how key ideas and practices have developed and are contested), offering a more comprehensive understanding of the scientific landscape of doping prevalence estimation.

### 2.2. Literature Search and Study Selection

**Figure A1:**
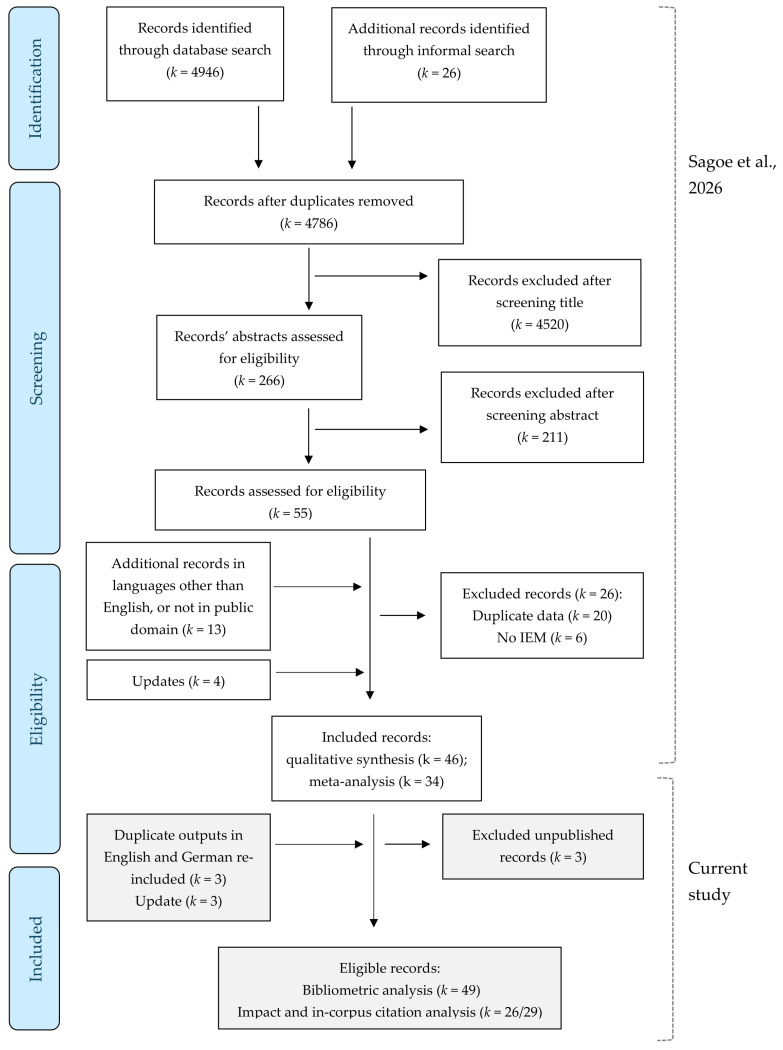
PRISMA flow diagram (Sagoe et al., 2026 [50]) updated with post-2023 records (April 2026).

The present manuscript and the companion review (Sagoe et al., 2026 [50]) derive from the same PROSPERO-registered, PRISMA-aligned search and screening workflow. To keep this paper self-contained, the procedures below mirror those reported in Sagoe et al. [50], with the only modification being an extension of the search beyond 2023 that yielded three additional eligible studies. The updated PRISMA flow diagram is provided in Appendix A (Figure A1).

#### 2.2.1. Protocol and Registration

The study corpus was assembled via a PROSPERO-registered (CRD42022373691) systematic search and screening process (PRISMA-aligned), which underpins both the present manuscript and the companion review (Sagoe et al. [50]). Although this manuscript reports a bibliometric and interpretive synthesis, the underlying corpus was identified using systematic search and screening procedures to ensure transparency and reproducibility.

#### 2.2.2. Information Sources

We conducted systematic searches in the following databases (English): ProQuest, PsycNET, PubMed, Web of Science, and Google Scholar. To capture German-language outputs, we searched SPORTDiscus, SPONET, BISp-Surf, Scopus, Web of Science, and Google Scholar using German-language equivalents of the English search terms. In addition, automated searches were conducted in French, Russian, and Spanish to identify relevant outputs in those languages. To enhance comprehensiveness, we also searched OpenGrey (SIGLE) and screened reference lists of included studies and relevant reports.

#### 2.2.3. Eligibility Criteria and Study Selection

Studies were eligible if they used indirect estimation models (randomised and non-random) to estimate doping prevalence in sport and were published in English, Dutch, German, French, Russian, or Spanish.

Records from all sources were collated, and duplicates were removed. Titles and abstracts were screened against the eligibility criteria, followed by full-text assessment of potentially relevant records. Reasons for exclusion were recorded at the full-text stage in line with PRISMA [57]. Any uncertainties were resolved through discussion within the author team.

#### 2.2.4. Data Extraction

Using a standardised extraction form, we extracted the following from each included study: author(s) and publication year; model used; estimated doping prevalence; and noncompliance assessment. Consistent with a content-analytic approach, DS conducted the initial data extraction and eligibility-based selection [50]. An updated search and data extraction from the additional studies were conducted by AP.

#### 2.2.5. Search Extension and Update

For the April 2026 update, Dimensions.ai and Google Scholar were searched using targeted combinations designed to retrieve IEM-specific outputs, including queries structured as follows: (specific IEM name) AND doping AND sport AND prevalence. To reduce retrieval of non-source material, citations were excluded from Google Scholar screening, and the results were limited to documents with accessible bibliographic information sufficient for eligibility assessment and data extraction.

Beyond these structured searches, the research team has maintained an ongoing, domain-specific surveillance of the IEM-based doping prevalence literature (including monitoring the emergence of new IEM approaches) since 2011. This expert-curated collection was used solely to support supplementary identification (e.g., cross-checking completeness and flagging newly released outputs). As such, it informed but did not replace the database searches or alter the prespecified eligibility criteria.

### 2.3. Data

The updated corpus comprises 49 eligible outputs. Three outputs (two confidential research reports and one unpublished manuscript) included in the systematic review by Sagoe et al. [50] are not listed here because they constitute grey literature with limited public accessibility. As these items are not readily retrievable and were not used in the bibliometric mapping or critical reflection, they were excluded from Table 1 for transparency and comparability. The remaining 49 outputs were qualitatively analysed, and a subset of publications indexed in the Web of Science (*k* = 26) and/or Scopus (*k* = 29) databases were eligible for bibliometric analysis (Table 1). The latest database literature check for update was conducted in April 2026.

For indexed outputs, primary focus was determined based on the study’s stated aims and the framing of its discussion. To ensure comparability with WoS and Scopus classifications, only outputs with a DOI and formal database indexing were classified. Research reports, book chapters, magazine articles, and outputs not indexed in either database were included in the qualitative synthesis but were not assigned a classification category.

**Table 1:** Outputs reporting doping prevalence estimates using IEMs, listed in alphabetical order.

References	Year	Publication Language	Type of Output	Primary Focus	WoS	Scopus
Abdulrazzaq and Tareq [58]	2023	English	academic journal	applied	no	no
Backhouse et al. [59]	2016	English	research report	applied	no	no
Balk and Dopeide. [60]	2021	Dutch	research report	applied	no	no
Balk et al. [61]	2023	English	academic journal	applied	no	no
Boardley et al. [62]	2019	English	academic journal	applied	yes	yes
Breuer and Hallmann [63]	2013	German	monograph	applied	no	no
Christiansen et al. [64]	2023	English	academic journal	applied	yes	yes
Cruyff et al. [65]	2024	English	academic journal	method	yes	yes
Dietz et al. [66]	2013	English	academic journal	applied	yes	yes
Dietz et al. [67]	2016	English	academic journal	applied	yes	yes
Duiven and de Hon [68]	2015	Dutch	research report	applied	no	no
Elbe and Pitsch [69]	2018	English	academic journal	applied	no	yes
Fincoeur and Pitsch [70]	2017	Dutch	academic journal	applied	no	no
Franke et al. [71]	2017	German	academic journal	applied	yes	yes
Frenger et al. [72]	2016	English	academic journal	applied	yes	yes
Heller et al. [73]	2020	English	academic journal	applied	yes	yes
Heyes [74]	2022	English	PhD thesis	applied	no	no
Hilkens et al. [75]	2021	English	academic journal	applied	yes	yes
James et al. [76]	2013	English	academic journal	method	yes	yes
Nakhaee et al. [77]	2013	English	academic journal	applied	no	no
Nilaweera et al. [78]	2020	English	conference abstract	applied	no	no
Petróczi et al. [79]	2022	English	academic journal	method	yes	yes
Pitsch [80]	2018	English	book chapter	applied	no	no
Pitsch [81]	2022	English	academic journal	applied	yes	yes
Pitsch and Christiansen [82]	2026	English	academic journal	applied	yes	yes
Pitsch and Emrich [83]	2012	English	academic journal	applied	yes	yes
Pitsch et al. [84]	2005	German	magazine	applied	no	no
Pitsch et al. [85]	2007	English	academic journal	applied	no	yes
Pitsch et al. [86]	2009	German	book chapter	applied	no	no
Pitsch et al. [87]	2009	German	magazine article	applied	no	no
Pitsch et al. [88]	2009	English	book chapter	applied	no	no
Pitsch et al. [89]	2013	German	book chapter	applied	no	no
Plessner and Musch [90]	2002	German	book chapter	applied	no	no
Reiber et al. [91]	2022	English	academic journal	method	yes	yes
Robach et al. [92]	2024	English	academic journal	applied	no	yes
Sayed et al. [93]	2022	English	academic journal	method	yes	yes
Sayed et al. [94]	2024	English	academic journal	method	yes	yes
Sayed et al. [95]	2024	English	academic journal	method	yes	yes
Sayed et al. [96]	2026	English	academic journal	method	yes	yes
Schröter et al. [97]	2016	English	academic journal	method	yes	yes
Schu and Haller [98]	2025	English	academic journal	applied	yes	yes
Seifarth et al. [99]	2019	English	academic journal	applied	yes	yes
Simon et al. [100]	2006	English	academic journal	applied	yes	yes
Stamm et al. [101]	2011	German	academic journal	applied	no	no
Striegel [102]	2012	German	book chapter	applied	no	no
Striegel et al. [103]	2010	English	academic journal	applied	yes	yes
Stubbe et al. [104]	2014	English	academic journal	applied	yes	yes
Ulrich et al. [105]	2018	English	academic journal	applied	yes	yes
Ulrich et al. [34]	2023	English	academic journal	method	yes	yes

### 2.4. Data Analysis

Data analysis comprised two components, including a critical assessment and bibliometric mapping, followed by an integrative synthesis of findings from both approaches.

#### 2.4.1. Critical Assessment

Critical appraisal was conducted to provide a conceptual and methodological context for the development of IEMs in doping prevalence research. This component aimed to synthesise the theoretical rationale, model evolution, and methodological debates underpinning the use of IEMs, thereby situating the bibliometric findings within the broader scientific and applied discourse. This review is based on the same body of literature identified in the companion systematic review and meta-analysis [50], updated and supplemented by additional methodological and conceptual papers that informed the historical and theoretical development of IEMs. Each article was examined for its contribution to the conceptual understanding or methodological refinement of IEMs in the context of sensitive or transgressive behaviour research, focusing on (1) the rationale for using IEMs in doping studies, (2) variations in model implementation and interpretation, (3) common methodological challenges such as instruction compliance, and (4) emerging solutions, including model extensions and cheating-detection variants.

#### 2.4.2. Bibliometric Analysis

Bibliometric mapping involved examining temporal trends in outputs, authors and authors’ institutional affiliations, dominant outlets (journals), and fields where doping prevalence estimation studies were presented. Research fields and topics in WoS and Elsevier’s SciVal were catalogued and analysed for dominant patterns. Academic impact was assessed via time-normalised citation recorded in Web of Science (WoS), as well as from Scopus’ Field-Weighted Citation Impact (FWCI) and SciVal Topic Prominence, which is a composite indicator that ranks a research topic’s momentum by combining recent citation counts, Scopus view counts, and the average CiteScore of the journals in which the topic’s papers appear. Citation analysis in this paper retained the conventional bibliographic details found in traditional citation indices and augmented them with additional contextual information, including the citation statement, its surrounding context, and the location of the citation within the citing article [106,107,108,109]. In addition to examining citations at the level of individual articles, we also analysed studies according to the type of citation. To enhance the model’s explanatory power, two additional features were incorporated. Nodes (outputs) were classified into categories based on the specific IEM employed in each article, and edges (citation links) were categorised according to the role of the citation, whereby the citation statements represented by the links. The taxonomy of these roles was simplified into four categories: (1) method—where the cited article was used for methodological purposes only; (2) multiple use—where the citation served several purposes (e.g., methodological reference and conceptual discussion); (3) other—encompassing non-central functions such as brief mentions; and (4) secondary data analysis. Additionally, author overlap between cited and citing papers was examined to account for self-citation and collaborative influence.

*Latent community structures* were explored with network maps. First, we constructed a network map of all authors associated with the included outputs. Clusters were identified using the Louvain method implemented in the igraph package v2.2.3 for R [110]. To evaluate the extent to which the included outputs form a coherent line of research, a local citation network model was applied. This model represents the network of citations among the included studies by considering both incoming and outgoing citations restricted to this set of papers. The background and interconnectedness of the selected papers in terms of research communities were evaluated in a co-document network based on shared authorships. This model is conceptually the inverse of a conventional co-author network, meaning that rather than connecting authors who have written together, it connects papers that share one or more authors. In this framework, two papers (A and B) are linked if they have at least one common author. By focusing on publications rather than individuals, the co-document network captures the intellectual structure of the field through patterns of shared authorship. This approach is particularly useful for identifying research communities or ‘intellectual camps’ assessing the cohesiveness of the literature, and detecting potential bridging papers that connect otherwise separate groups. In the present study, the co-document network was applied to explore how studies employing IEMs in doping research cluster around shared expertise, methodological preferences, and research focus (topics). We applied the Louvain algorithm to detect coherent subgraphs representing clusters of closely related publications [111].

Network visualisation and interpretation were carried out to aid the qualitative understanding of the bibliometric structures and to facilitate the interpretation of how methodological preferences, collaborative patterns, and intellectual lineages structure the field of doping prevalence estimation with IEMs. The resulting co-authorship network and citation network were visualised using Cytoscape web 1.0.5 (www.cytoscape.org, accessed on 20 April 2026). Co-document networks were visualised using force-directed layouts, which position nodes based on the strength and density of their connections.

#### 2.4.3. Assessment of Overall Evidentiary Strength

Overall evidentiary strength was assessed using a modified version of the framework proposed by Palmateer et al. (p. 846) [112], adapted for IEM-based doping prevalence research. Prevalence estimates were grouped into 5% bins and cross-tabulated by IEM. Cells recorded the number of studies and were coded by adjustment for survey instruction noncompliance (adjusted vs. unadjusted) and by analytical status (primary vs. secondary). Cumulative evidence was qualitatively interpreted as sufficient, tentative, insufficient, or none, based on the convergence and robustness of available primary studies.

#### 2.4.4. Data Integration

Following the principles of the convergent parallel design [56], the bibliometric, narrative, and evidentiary assessment components were conducted and analysed independently, with results integrated during the interpretation phase. Quantitative findings from the bibliometric mapping, such as publication trend over time, outlets, research topic classifications, citation structures, and collaboration networks, were compared and cross-referenced with qualitative insights from the narrative synthesis, including theoretical debates, methodological adaptations, and conceptual developments. Integration was achieved through interpretive triangulation, allowing complementary strands of evidence to inform each other.

## 3. Results

**Figure 2:**
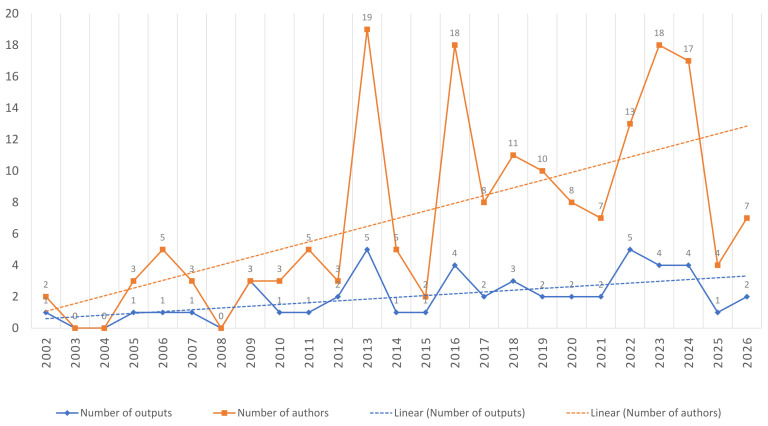
Temporal trends in the use of IEM for estimating doping prevalence in the number of published outputs and contributing authors.

The number of published studies increases only gradually over the observed period, indicating a relatively slow expansion of the evidence base, with the number of outputs fluctuating between two and five per year (see Figure 2). In contrast, the number of unique researchers involved shows a more pronounced upward trend. This divergence suggests that, while growth in outputs remains modest, IEM-based doping prevalence estimation is attracting an increasingly broader research community, pointing to a slowly rising methodological interest and collaborative engagement beyond what is reflected by publication counts alone.

### 3.1. Publication Patterns

**Figure 3:**
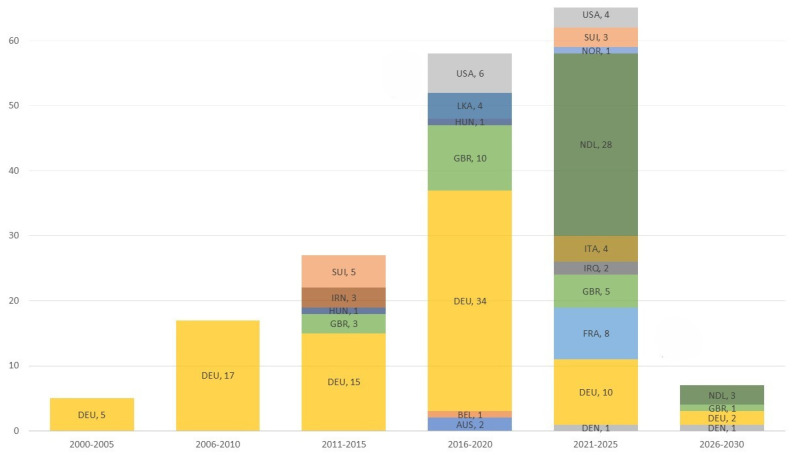
Trends in the use of IEMs for doping prevalence research by author diversity across countries. Numbers are author appearances by their respective nationality (denoted by different colours), allowing for the same author to appear multiple times on multiple outputs.

Among the 49 retained records, outputs were dominantly research articles (*k* = 33), followed by book chapters and monographs (*k* = 7), publicly available reports (*k* = 3), published conference abstracts (*k* = 2), magazine articles (*k* = 3), and a PhD thesis (*k* = 1). Early adoption and applications of IEMs to estimate doping prevalence can be observed by authors from Germany (see Figure 3) since 2002. The only other countries where researchers demonstrated sustained involvement in studies with IEMs were the UK and the Netherlands, including both national and international collaborations.

Most outputs were published in English (*k* = 38), followed by German (*k* = 8) and Dutch (*k* = 3), with some overlap and duplication between English- and German- and English- and Dutch-language versions. These instances represent duplicate publications, where identical datasets and results were disseminated across multiple outputs. Specifically, two German-language studies [86,87] reported the same data later published in English by Pitsch and Emrich [83] and also included material from an earlier investigation [84] that was subsequently re-presented in another publication [85]. Results from a recent Dutch doping prevalence study [60,68] were republished in Balk et al. [61]. Pitsch [81] and Christiansen et al. [64] reported identical data, with further subgroup analysis presented in Pitsch and Christiansen [82].

A second category comprised secondary analyses, where data were re-analysed using refined algorithms or alternative assumptions. Ten studies in three sets fell into this category. Ulrich et al. [105] and Petróczi et al. [79] were conducted in the same setting and shared one dataset but applied different IEM variants. Reiber et al. [91] and Ulrich et al. [34] subsequently re-analysed the same data generated with UQM [105] and SSC [79] models, respectively, testing a different hypothesis and introducing revised assumptions about the magnitude and causes of noncompliance with survey instructions. Likewise, data first reported by Cruyff et al. [65], where two sets of results were presented, including one set for the unadjusted prevalence estimation (assuming full compliance with survey instructions) and a set adjusted for self-protective responding, were later re-examined in Sayed et al. [96] to assess the potential impact of random responding (i.e., participants accelerating through the survey by selecting responses at random). The third set revolved around the Kuk’s model and comprised two parent studies [68,75] and two subsequent re-analyses to investigate the impact of timeframe reference (i.e., lifetime (ever) and current (last year)) and evasive responding [94,95].

### 3.2. Publication Channels and Research Fields

The selected studies are distributed across a wide range of publication outlets and research fields (Tables S1 and S2), reflecting substantial dispersion despite a shared substantive focus. Of the 33 journal outputs identified with DOIs (26 are indexed in Web of Science and 29 are in Scopus). Twelve outputs were concentrated in just three journals (PLOS One, Sports Medicine/Sports Medicine–Open, and Performance Enhancement & Health), while the remaining 22 appeared in 22 different outlets. Although 18 of the 26 journals were ranked in the top quartile (Q1) of the Scimago Journal Ranking, this dispersion suggests that IEM-based doping prevalence research has been evaluated by diverse peer-review communities with varying levels of methodological expertise. Notably, only about half of the journals targeted a sports science readership, indicating that doping prevalence often serves as a test case for methodological development rather than the sole focus of inquiry.

**Figure 4:**
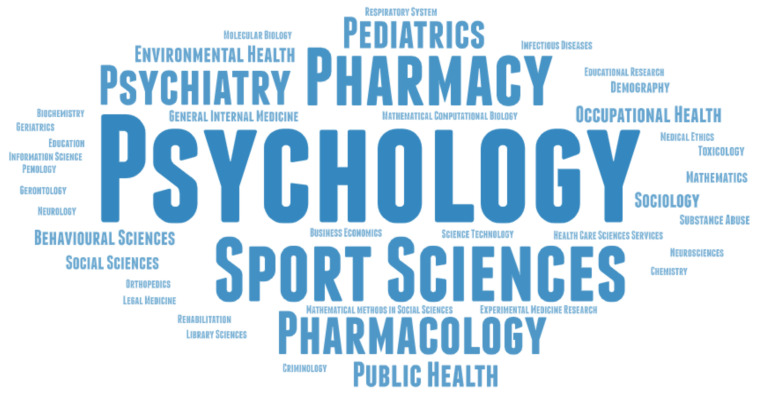
Word cloud of Web of Science subject categories, where font size reflects category prominence (i.e., the more frequently a study is assigned to a given subject area, the larger its label appears).

Disciplinary clustering aligns with this pattern. Sports science and psychology journals tend to prioritise applied prevalence estimates, whereas statistical and methodological journals focus on model validation and analytical refinement, reinforcing the interdisciplinary yet fragmented nature of the field. WoS subject categorisation further amplifies this dispersion. Although all 26 empirical studies examined doping prevalence in sport, they are indexed across 34 subject categories in WoS (Figure 4), giving the appearance of a broad evidence base despite substantial overlap in data, models, and author networks.

At its core, the field is anchored in ‘psychology’, ‘sports science’, and ‘public, environmental, and occupational health’, framing doping primarily as a sport-related behavioural health issue. Additional classifications in ‘psychiatry’ and ‘substance abuse’ further accentuate a clinical framing, despite limited engagement with diagnosis or treatment. Methodologically driven categories such as ‘mathematics’ and ‘mathematical methods in the social sciences’ contribute disproportionately to the field’s visibility. Social science perspectives remain uneven, with ‘sociology’ moderately represented and ‘governance- or policy’-oriented fields largely marginal. Output-level categorisation is given in Supplementary Materials (Table S3).

Looking from a different angle, the outputs were distributed across six high-momentum SciVal Topics (algorithmically assigned by Scopus), with the largest concentrations found in ‘doping policies and athlete integrity in sports’ (*k* = 11/30) and ‘randomised response techniques for sensitive surveys’ (*k* = 11/30), reflecting both the centrality of doping-related concerns and the methodological advances used to estimate their prevalence. The remaining four topics comprise ‘research on the health risks of anabolic steroid use’ (*k* = 3), ‘prescription drug misuse and cognitive enhancement’ (*k* = 3), ‘nutritional supplement use and performance’ (*k* = 1), and ‘erythropoietin-related doping and detection methods’ (*k* = 1).

### 3.3. Framing of Doping in Titles and Publication Contexts

An analysis of the publication titles and journal outlets reveals clear patterns in how doping is conceptually framed across disciplines. These patterns mirror the disciplinary homes of the journals, highlighting how scientific communities construct the meaning and boundaries of doping and doping prevalence. Specifically, titles published in sports science and medicine journals (e.g., Sports Medicine, Scandinavian Journal of Science & Medicine in Sport, Journal of Sport Sciences) typically adopt an epidemiological and empirical framing, positioning doping as a measurable phenomenon. Terms frequently used in titles emphasise quantification, method, and comparability with terms such as ‘prevalence, estimation, frequency’, and ‘use’. This reflects a biomedical and sports science discourse, where doping is treated as a population-level health and/or integrity issue requiring methodological rigour and large-scale evidence.

In contrast, publications in journals such as Addiction, Drug and Alcohol Dependence, Performance Enhancement and Health, and Psychology of Sport and Exercise frame doping as a behavioural or psychosocial phenomenon. Here, the lexical field shifts from ‘prevalence’ to ‘use’, often in combination with ‘attitude’, ‘*susceptibility*’, or ‘vulnerability’, which suggests an interpretive stance oriented toward individual human problem behaviour rather than population measurement. A third cluster, comprising journals such as the International Review for the Sociology of Sport, the Journal of Criminal Law, and Criminology and Criminal Justice, adopts a moral, regulatory, or sociological framing, wherein doping appears as a social deviance or policy problem—embedded in wider issues of governance, integrity, and the health of elite sport systems. The recurring use of terms like ‘risk management’ and ‘sport-induced substance use’ reflects this more normative and institutional perspective.

### 3.4. Evidentiary Synthesis

The synthesis of IEM-based prevalence estimates reveals substantial heterogeneity across methods, samples, and analytical approaches (Table 2 and Table S4). To assess the strength of evidence within this diverse body of work, we consider the density of estimates falling into specific prevalence ‘bins’. The number of evidence points exceeds the number of unique studies because many publications report multiple estimates across subgroups, time points, or analytical specifications.

The overall picture in Table 2 shows that evidence is unevenly distributed across IEM families, reflecting shifts in methodological popularity over the past 25 years. While prevalence estimates span a wide range, consistent with variation in athlete populations and definitional differences, the strongest and most consistent concentration lies within the lower prevalence bins. Across methods, designs, and operationalisations, repeated clustering in the 0–20% range indicates a more stable and coherent empirical signal in this part of the distribution. In contrast, higher prevalence estimates appear less frequently and are more closely tied to specific methods, analytical assumptions, or a unique sample. This overall picture appears to be congruent with the more nuanced meta-analytical synthesis presented in Sagoe et al. [50], indicating sufficient evidence up to 25%. Higher prevalence estimates of near or above 50% appear to be inconclusive or derived from a single study with a small sample.

### 3.5. Scientific Impact

The average Field-Weighted Citation Impact (FWCI) of the included studies was 1.771 (*SD* = 2.429; median: 0.905; range: 0.00–10.38). Overall, the scientific impact of the corpus is above the international average, as the median value reaches the global field-normalised benchmark of 1.0, while the mean value exceeds it. The slightly lower median reflects a skewed distribution driven by a small number of highly cited outliers.

**Figure 5:**
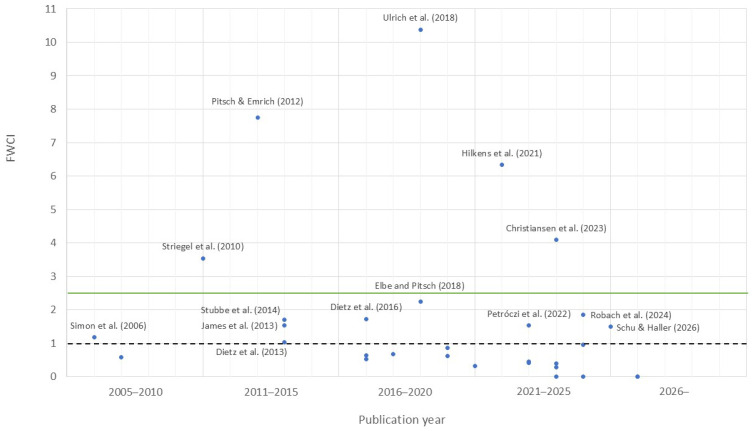
Scientific impact of the included studies, represented by their Field-Weighted Citation Index (FWCI) scores. Studies with FWCI < 1 are indicated but not individually labelled to preserve graph readability. Black dotted line marks the world average; green dotted line shows the trimmed mean citation score for the corpus. Ulrich et al. 2018 [105]; Pitsch & Emrich 2012 [83]; Hilkens et al. 2021 [75]; Christiansen et al. 2023 [64]; Striegel et al. 2010 [103]; Elbe and Pitsch 2018 [69]; Simon et al. 2006 [100]; Stubbe et al. 2014 [104]; Dietz et al. 2016 [67]; James et al. 2013 [76]; Petróczi et al. 2022 [79]; Robach et al. 2024 [92]; Schu & Haller 2026 [98]; Dietz et al. 2013 [66].

A more nuanced picture emerges when examining the temporal distribution of citation scores. Despite the intrinsic age normalisation of the MNCS metric, maintaining a three-year citation window remains advisable for reliable impact assessment. As illustrated in Figure 5, most outputs published between 2017 and 2025 cluster around or above the world average (1.0; black dotted line). Excluding the top and bottom 10 percent, the trimmed mean yields a still relatively high mean citation score of 1.53 (green dotted line), indicating that the field’s influence has been both sustained and robust over time.

Based on the Scopus data (Table S3), the dataset demonstrates sustained topical relevance and strong, albeit heterogeneous, citation performance across the major strands of anti-doping research. The Field-Weighted Citation Impact (FWCI) values of these topics show considerable variability, although several publications, particularly those within the most prominent topics, exceed the global citation average. Topic-level impact scores with a mean FWCI of 2.00 (*SD* = 2.71) for ‘randomised response techniques for sensitive surveys’ and 1.77 (*SD* = 2.47) for ‘doping policies and athlete integrity in sports’ suggest that the field is equally split between method development and its applicability in assessing the prevalence of doping in sport. The remaining four topics comprise research on the health risks of anabolic (and anabolic–androgenic) steroid use (*k* = 3, mean FWCI = 2.17, *SD* = 2.46), prescription drug misuse and cognitive enhancement (*k* = 3, mean FWCI = 1.99, *SD* = 2.77), nutritional supplement use and performance (*k* = 1, FWCI = 1.75), and erythropoietin-related doping and detection methods (*k* = 1, FWCI = 1.85).

### 3.6. Authors and Authorship

**Figure 6:**
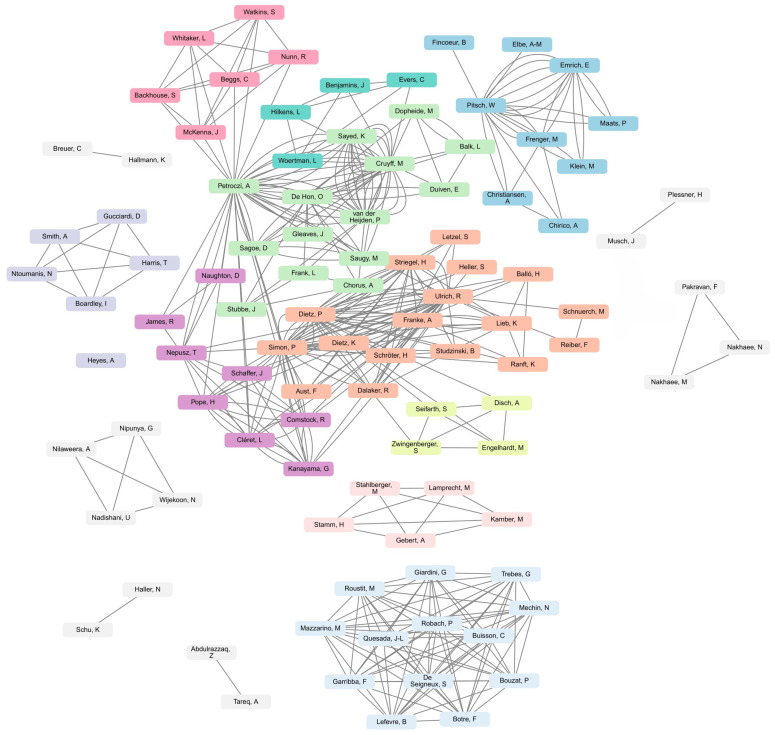
Co-authorship collaboration network among the included studies, with colours indicating empirically identified clusters.

One-hundred unique authors contributed to the literature on doping prevalence estimation with IEMs, collectively appearing 185 times (see Table S5). Among them, only 29 authors (29.0%) contributed more than one output, and only 12 authors (12.9%) had three outputs or more: Pitsch (*k* = 13), Simon (*k* = 9), Cruyff (*k* = 8), Petróczi (*k* = 8), Ulrich (*k* = 8), Van der Heijden (*k* = 7), Emrich (*k* = 7), Dietz (*k* = 7), Sayed (*k* = 6), Striegel (*k* = 5), De Hon (*k* = 4), and Frenger (*k* = 3). The collaboration pattern among the authors who have worked with IEMs to estimate doping prevalence, visible in co-authorships, offers an intriguing picture (Figure 6). Authors in the corpus formed two unconnected clusters of different sizes and six unconnected research groups. The small cluster is a tightly knit group of multiple jointly authored outputs centred around Pitsch. The large cluster is an amalgamation of three loosely connected groups around Ulrich and Cruyff, with Petróczi serving as a bridge between the other two.

Across the corpus, men were the majority among those developing, refining, or applying IEMs to doping prevalence, accounting for 71% of all authors. Gender imbalance was even more pronounced among lead contributors. Of the 48 authored outputs, 39 (81.2%) listed a male first author, and among the 44 outputs where a corresponding author could be identified (some research reports did not specify one), only 14 (31.8%) were women. Last authorship was not analysed due to varying disciplinary conventions in author order across the contributing fields. This observed gender imbalance is not merely an equality statistic but may carry epistemic implications for how research questions are framed and which methodological approaches are privileged [113].

### 3.7. Research Communities

**Figure 7:**
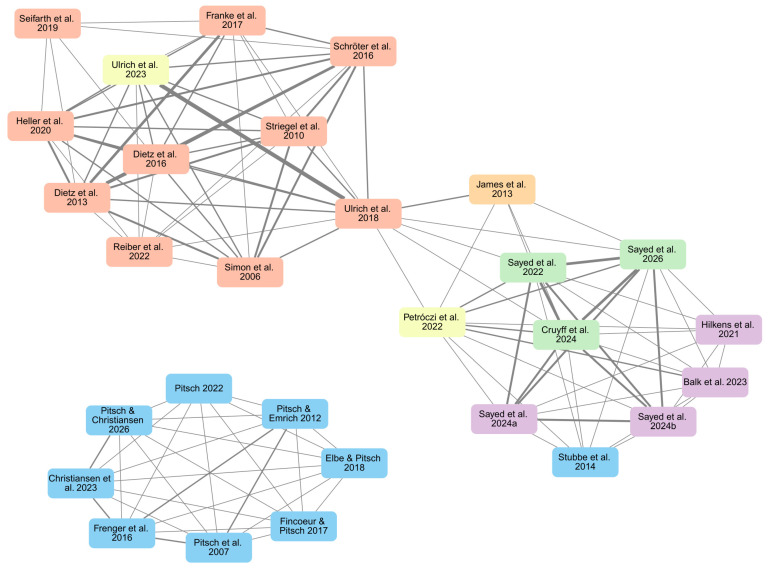
Co-document network colour-coded by the IEM used (blue: FR, green: ECWM, orange: SSC and UQM; peach: UQM; purple: Kuk’s model; yellow: SSC; pale peach: UQM and SSC); line thickness reflects the number of shared authors, with thicker lines indicating greater overlap between author groups. Balk et al. 2023 [61]; Christiansen et al. 2023 [64]; Cruyff et al. 2024 [65]; Dietz et al. 2013 [66]; Dietz et al. 2016 [67]; Elbe and Pitsch 2018 [69]; Fincoeur & Pitsch 2017 [70]; Franke et al. 2017 [71]; Frenger et al. 2016 [72]; Heller et al. 2020 [73]; Hilkens et al. 2021 [75]; James et al. 2013 [76]; Petróczi et al. 2022 [79]; Pitsch 2022 [81]; Pitsch & Christiansen 2026 [82]; Pitsch & Emrich 2012 [83]; Pitsch et al. 2007 [85]; Reiber et al. 2022 [91]; Sayed et al. 2022 [93]; Sayed et al. 2024a [94]; Sayed et al. 2024b [95]; Sayed et al. 2026 [96]; Schröter et al. 2016 [97]; Seifarth et al. 2019 [99]; Simon et al. 2006 [100]; Striegel et al. 2010 [103]; Stubbe et al. 2014 [104]; Ulrich et al. 2018 [105]; Ulrich et al. 2023 [34].

The co-document network based on shared authorship reveals the underlying structure of research communities contributing to IEM-based prevalence estimations applied to doping. The network, presented in Figure 7, displays a clear community organisation among the included outputs. For ease of interpretation, detected communities (sets of outputs) are colour-coded by clusters.

As Figure 7 shows, two distinct and unconnected components emerged. The smaller component represents a fully connected group centred around Pitsch, indicating a tightly knit collaboration network with limited external connections. The larger component is more complex, comprising two coherent but only loosely interconnected subgroups around Ulrich and Cruyff. These subgroups are linked through a bridging publication and, more precisely, through Petróczi—whose authorship on Ulrich et al. [105] connects the two otherwise separate clusters. This structural pattern mirrors the configuration observed in the overall author collaboration network (see Figure 6), suggesting that within-sample citation may be influenced more by existing collaborations and self-citation than by direct engagement with external scientific content. Notably, each community spans multiple publication years, indicating stable and sustained collaboration over time rather than short-term or project-specific partnerships. It can also be observed that certain author groups display a consistent preference for specific IEM variants. This pattern suggests that the selection of a model may not only be determined purely by rational or technical considerations—such as selecting the most appropriate tool for a given research question or population—but also by familiarity, available expertise within the research team, and the legacy of prior collaborations. Language, training background, and beliefs about the truth (i.e., what is ‘true’ prevalence) and the best or most valid model appear to reinforce these preferences.

**Figure 8:**
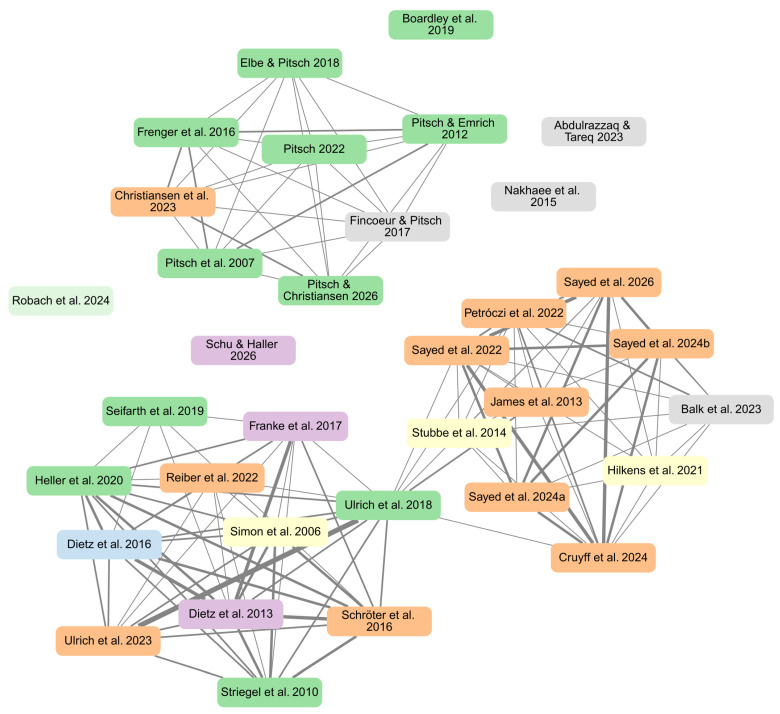
Co-document network colour-coded according to Scopus SciVal topics (green: doping policies and athlete integrity in sports; orange: randomised response techniques for sensitive surveys; purple: prescription drug misuse and cognitive enhancement, yellow: research on the health risks of anabolic steroid use; pale green: erythropoietin-related doping and detection methods; grey: not in SciVal; blue: nutritional supplement use and performance). Abdulrazzaq and Tareq [58]; Boardley et al. 2019 [62]; Christiansen et al. 2023 [64]; Cruyff et al. 2024 [65]; Dietz et al. 2013 [66]; Dietz et al. 2016 [67]; Elbe and Pitsch 2018 [69]; Fincoeur & Pitsch 2017 [70]; Franke et al. 2017 [71]; Frenger et al. 2016 [72]; Heller et al. 2020 [73]; Hilkens et al. 2021 [75]; James et al. 2013 [76]; Nakhaee et al. [77]; Petróczi et al. 2022 [79]; Pitsch & Christiansen 2026 [82]; Pitsch & Emrich 2012 [83]; Pitsch 2022 [81]; Pitsch et al. 2007 [85]; Reiber et al. 2022 [91]; Robach et al. 2024 [92]; Sayed et al. 2022 [93]; Sayed et al. 2024a [94]; Sayed et al. 2024b [95]; Sayed et al. 2026 [96]; Schröter et al. 2016 [97]; Schu & Haller [98]; Seifarth et al. 2019 [99]; Simon et al. 2006 [100]; Striegel et al. 2010 [103]; Stubbe et al. 2014 [104]; Ulrich et al. 2018 [105]; Ulrich et al. 2023 [34]; Balk et al. 2023 [61].

Overlaying Scopus’ algorithmically generated SciVal Topics, derived from citation patterns and co-document relationships (see Supplementary Table S3 for output-level classification), onto the co-document network provides a more nuanced view of the literature landscape. Consistent with Figure 6, which shows a coherent overlap between clusters and IEMs employed, two dominant SciVal topic clusters also emerged (Figure 8). One cluster centres on doping prevalence research within the topic ‘doping policies and athlete integrity in sports’ and is largely associated with the work of Pitsch and colleagues. The other focuses on methodological innovation within ‘randomised response techniques for sensitive surveys’, with key contributions from Cruyff, Sayed, and Petróczi. The remaining publications and authors are distributed across four additional SciVal Topics, reflecting the diversity of research directions within the field.

The close correspondence between our content-based classification and Scopus-assigned SciVal Topics provides an important validity check for the analytical framework used in this review. Notably, all but one output (Christiansen et al. [64]) classified under ‘randomised response techniques for sensitive surveys’ had a primary focus on method development, methodological refinement, or model testing (Table 1 and Table S3), indicating strong convergence between externally generated citation-based topic modelling and researcher-led classification based on stated study aims. The single exception represents an applied study that deployed an established IEM in a recreational sport context without a substantive method development component, suggesting that its inclusion within this SciVal Topic likely reflects keyword usage or citation proximity rather than methodological innovation *per se*. Importantly, the relatively small and well-defined corpus examined here enabled manual classification and direct comparison with algorithmically derived topic assignments. The observed close match suggests that SciVal Topics capture meaningful distinctions in the intellectual organisation of the field, particularly the separation between method-centric research and applied prevalence estimation within doping studies, and supports the use of such automated classifications in future large-scale bibliometric analyses of anti-doping research, where manual processing is not feasible.

This positional mapping is important because it highlights the conceptual role of each body of work. Studies primarily concerned with methodological development, refinement, and validation often generate prevalence estimates as secondary outputs of their analyses. These values should therefore be interpreted with caution. Using isolated or selectively extracted figures from method-focused studies as direct evidence of doping prevalence risks misrepresenting the intended scope and limitations of the research.

### 3.8. Local Citation Network

**Figure 9:**
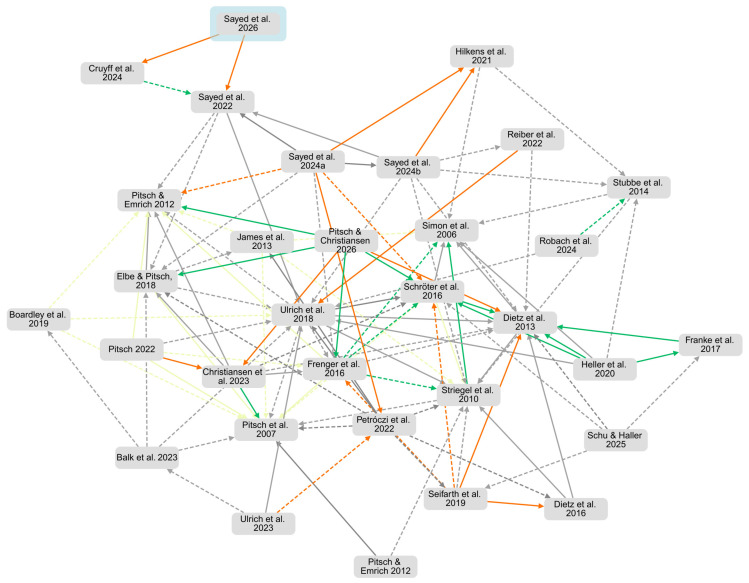
Within-corpus citation network, with edge colours denoting citation type (green = method; yellow = multiple use; grey = other; orange = data/secondary analysis). Solid lines indicate shared authorship between citing and cited outputs; dashed lines indicate no overlapping authorship. Balk et al. 2023 [61]; Boardley et al. 2019 [62]; Christiansen et al. 2023 [64]; Cruyff et al. 2024 [65]; Dietz et al. 2013 [66]; Dietz et al. 2016 [67]; Elbe and Pitsch 2018 [69]; Franke et al. 2017 [71]; Frenger et al. 2016 [72]; Heller et al. 2020 [73]; Hilkens et al. 2021 [75]; James et al. 2013 [76]; Petróczi et al. 2022 [79]; Pitsch & Christiansen 2026 [82]; Pitsch & Emrich 2012 [83]; Pitsch 2022 [81]; Pitsch et al. 2007 [85]; Reiber et al. 2022 [91]; Robach et al. 2024 [92]; Sayed et al. 2022 [93]; Sayed et al. 2024a [94]; Sayed et al. 2024b [95]; Sayed et al. 2026 [96]; Schröter et al. 2016 [97]; Schu & Haller [98]; Seifarth et al. 2019 [99]; Simon et al. 2006 [100]; Striegel [102]; Striegel et al. 2010 [103]; Stubbe et al. 2014 [104]; Ulrich et al. 2018 [105]; Ulrich et al. 2023 [34].

The local citation network model was used to evaluate the extent to which the included studies form a coherent line of research (Figure 9). The most informative feature of the network is that it comprises a single connected component, indicating that all articles in the sample are directly or indirectly linked through citation relations, with no isolated nodes. Beyond this overall connectedness, network-level measures show that the distances within the graph are small and the network is relatively compact overall (average shortest-path length = 2.16), suggesting that ideas and methods diffuse across the literature in just a few intermediary citations, with early method papers acting as hubs and recent statistical developments forming the far end of the knowledge chain. The diameter (d) of the network is four, meaning that the shortest path between any two papers involves only four citation links (e.g., Simon et al. 2006 [100] → Striegel et al. 2010 [103] → Ulrich et al. 2018 [105] → Sayed et al. 2022 [93] → Cruyff et al. 2024 [65]). Together, these structural features describe a highly cohesive research line in which successive studies display continuous awareness of prior work in the field.

Several core papers, such as Pitsch et al. [85], Striegel et al. [103], Ulrich et al. [105], and Dietz et al. [67], serve as key reference points for later outputs, forming the backbone of the citation network. To further explore the semantics of citation flow beyond structure, we refined the local citation network by incorporating the type of citation relationship. Node colours represent the IEM applied for prevalence estimation, while edge colours denote citation types. The taxonomy of citation roles was simplified into three categories: method, multiple use, and other mentions. Although these categories differ conceptually, the vast majority of citations fell under the mentioning type (acknowledging another study without direct relevance), while substantive method and multiple-use citations were treated as indicators of knowledge transfer.

The network depicted in Figure 9 exhibits both structural cohesion and functional connectivity. The citation flow consists predominantly of strong links, indicating that individual studies tend to engage with the methods, findings, or assumptions of preceding work. Importantly, these strong links do not necessarily imply endorsement because critiques and refinements can also generate dense citation connections. Based on author patterns, strong links within clusters are more likely to reflect methodological continuation and knowledge transfer, whereas links between clusters represent critical comparison or methodological debate.

When incorporating information about the specific IEM employed, an even more granular picture emerges. Two models dominate the corpus: the FR and UQM. The most frequent citation connections occur within these same-model pairings (FR → FR; UQM → UQM). However, cross-model links are also common, suggesting a degree of methodological awareness and continuity across the research community. Given the technical complexity of IEMs, the emergence of entirely new research groups without prior collaboration or co-authorship is rare, and when it happens, it tends to be a one-off research enterprise.

Secondary data analysis occurred only when researchers conducting the re-analysis had been involved, in some capacity, in the original data collection or primary analysis. Such involvement was recognisable either through overlapping authorship, indicating shared research communities across studies, or through contributions made via commissioned work, which may not appear in the citation network but were explicitly acknowledged in the publications. Across the corpus, no instances were identified in which an entirely independent research team reused or re-analysed data generated by others.

### 3.9. Network Cohesion, Weak Ties, and Brokerage

Nearly half of all citation links exhibit overlapping authorship. Nearly half of the 110 links (49, 44.5%) involved at least one author appearing on both the citing and cited papers. Within the largest component, two prominent subgroups are visible: a densely connected cluster centred on Striegel and Dietz (frequently linked with Ulrich) and a looser constellation around Petróczi, Sayed, and Stubbe. This pattern indicates a small expert base and repeated team-level collaborations. These self-referential patterns reinforce the presence of ‘invisible colleges’—informal, cohesive communication circles that organise knowledge flows within specialties and shape the growth of research areas [114,115].

From a network-theoretic perspective, the configuration we observe, namely dense internal linkages with selective cross-cluster connectors, aligns with classic theories of diffusion via weak ties [116] and brokerage across structural holes [117,118]. In such structures, a small number of bridges (e.g., citations linking Striegel et al. [103] to Ulrich et al. [105] and to Petróczi et al. [79]) carry ideas between otherwise segregated subgroups, enabling cross-fertilisation that dense intra-cluster ties alone cannot deliver. Taken together, tight intra-cluster linkages appear to enhance conceptual coherence and speed method transfer within the two main camps, yet their concentration within closely connected teams also risks insularity, potentially limiting cross-paradigmatic exchange and slowing broader theoretical integration, which is a trade-off long noted in studies on invisible colleges, weak ties, and structural holes [114,115,116,117,119].

## 4. Integrated Results and Narrative Insights

Against the rich literature on IEMs, spanning over half a century [20,21,120], their application to doping only began around the turn of the millennium [84,90], with the first full publication in English appearing in 2006 [100]. Our findings indicate limited variability in study origin, with the majority of studies included in the meta-analysis conducted in European countries. Bibliometric analyses revealed that this trend was primarily driven by the dominance of two closely linked but distinct research groups in Germany. Over time, however, the trends show the emergence of new research groups in the United Kingdom and the Netherlands. WADA’s establishment of a Working Group on Prevalence of Doping in Sport (2017–2023), with its focus on survey development [121], also facilitated the observed expansion in outputs, authorship, and diversity of IEM applications. Preferences for specific models are notable. For instance, the research group led by Ulrich predominantly applies the UQM, while Pitsch and colleagues favour the FR model. The CM and its variants have gained recognition in the field since being adopted by WADA’s working group, leading to a series of field testing [65,93] and methodological refinements over recent years [65,96,122].

Research regarding indirect estimations of doping prevalence is a field that is methodologically innovative yet structurally fragmented, with important implications for how doping prevalence estimates are produced, circulated, and interpreted. Combining quantitative indicators (e.g., publication patterns, outlets, and temporal trends) with qualitative analysis of research aims and framing provides a multidimensional picture of the intellectual development and epistemic orientation of IEM-based doping prevalence research. Across its development, the thematic focus of the field has shifted markedly. Early studies between 2006 and 2012 were primarily concerned with demonstrating the feasibility of indirect methods, most notably RRT, for estimating hidden doping behaviour in elite and fitness sport contexts. These contributions were typically framed as proof-of-concept studies aimed at showing that indirect questioning could yield plausible prevalence estimates where direct approaches failed. During the subsequent period from 2013 to 2019, the field expanded both empirically and conceptually.

Researchers increasingly embedded prevalence estimation within broader behavioural frameworks, examining gateway hypotheses, cognitive doping, and supplement use, while also extending empirical attention beyond elite sport to recreational and sub-elite populations. This phase was characterised by greater methodological experimentation, including the comparative application of multiple indirect techniques within the same samples. From around 2020 onwards, a pronounced methodological turn is evident. Recent studies increasingly focus on the development, critique, and refinement of IEMs themselves, with explicit attention to sources of bias, evasive responding, instruction noncompliance, and potential inflation effects. This has been accompanied by the re-analysis and re-interpretation of earlier, high-profile prevalence estimates in light of new empirical and analytical insights. Collectively, these developments signal a shift in the field’s core question from “how prevalent is doping?” toward “how trustworthy and interpretable are our estimates?”, with the latter giving way to method-driven, nuanced re-analyses that take noncompliance into account for improved validity.

Bibliometric patterns in publication outlets further reinforce this interpretation. The literature is highly dispersed across journals, but clusters around the four broad domains of sport and exercise medicine/sports science, behavioural science and psychology, methodological and statistical journals, and public health or substance-use outlets. Sports science and sports medicine journals typically publish event-based or elite athlete prevalence studies, while behavioural and social science journals emphasise the issues of sensitive behaviour, social desirability, and response processes. Methodological journals are largely devoted to model development and validation rather than substantive prevalence estimation (albeit producing prevalence estimations as a ‘by-product’ of model testing), whereas public health and addiction journals feature more prominently in early work and studies of fitness or recreational sport. This dispersion reflects considerable methodological sophistication but weak disciplinary consolidation, with parallel research communities that are only partially connected. Although the populations studied have diversified over time from elite athletes to recreational, fitness, and ultra-endurance athletes, elite sport continues to function as the dominant normative reference point for interpretation and policy relevance.

Geographically, the field remains predominantly European, driven in particular by German and Dutch research groups. Contributions from outside Europe are comparatively rare and tend to be a one-off context-specific application rather than programmatic. While this reflects Europe’s leading role in both methodological innovation and anti-doping policy, it also exposes a Eurocentric bias that limits the cultural and linguistic diversity of the evidence base. The framing of sensitive questions such as doping [50], together with respondents’ trust in researchers or institutions, is likely shaped by cultural and linguistic context. Although no studies to date have directly confirmed or refuted this assumption in sport, evidence from research outside sport suggests that social and cognitive cultural patterns play a fundamental role in shaping trust in sensitive surveys [123,124,125,126,127,128]. Cultural dimensions, social desirability pressures, and normative orientations such as modesty and honour interact to influence disclosure decisions, response styles, and perceptions of survey credibility and trustworthiness [129,130,131]. Limited participation from non-European regions may therefore restrict understanding of how IEM-based instruments should be adapted to diverse populations to ensure conceptual, ethical, and linguistic equivalence.

Our bibliometric and narrative analyses identified several instances in which identical datasets and findings were disseminated across multiple publications, sometimes in different languages or formats. Although such practices may increase accessibility, they complicate evidence synthesis by increasing the likelihood of double-counting and by artificially amplifying measures of scholarly impact. Re-analyses were most commonly motivated by efforts to refine IEM-based prevalence estimation and to model alternative patterns of survey instruction noncompliance. Consequently, multiple prevalence estimates are frequently reported from the same underlying samples. A clear example is the sequence of studies by Cruyff et al. [65] and Sayed et al. [96], which progressively extended the CM to account for self-protective responding and inattentive random responding, respectively. Similarly, Ulrich et al. [34] re-analysed data originally reported by Petróczi et al. [79], producing substantially different prevalence estimates and contributing to an ongoing methodological debate regarding the interpretation of earlier findings derived from the same populations and events [34,79,105].

Although these analytical refinements are scientifically defensible, they generate multiple, sometimes divergent estimates from identical datasets, complicating public-facing communication about doping prevalence. This challenge is evident in recent scholarly exchanges concerning the interpretation and policy relevance of such estimates [132,133,134]. While scenario-based modelling of noncompliance enhances methodological insight and empirical testability, interpretation depends critically on understanding the underlying behavioural assumptions and model specifications. Absent from this contextualisation, successive refinements may appear inconsistent or even suspect to researchers, policymakers, regulators, and media audiences, and thus, they may undermine confidence in IEMs and erode trust among practitioners who rely on prevalence estimates for risk assessment, adjudication, and evaluation of anti-doping policy effectiveness.

Taken together, the temporal and bibliometric evidence points to a clear epistemic evolution. An initial phase of estimation optimism and replication gave way to growing awareness of construct overlap and potential inflation, followed more recently by a period of reflexivity marked by bias modelling, uncertainty, and reassessment of legacy estimates. Later studies increasingly foreground model assumptions, researcher degrees of freedom, and interpretive limits, explicitly challenging the treatment of prevalence figures as stable or definitive indicators. This trajectory shows both the scientific maturation of the field and the persistent difficulties surrounding the communication and use of IEM-based doping prevalence estimates beyond specialist audiences.

Across the corpus, doping functions as a metonym for multiple overlapping phenomena, ranging from elite rule violations to everyday enhancement behaviours. A number of output titles signal this conceptual fluidity by referring interchangeably to doping, performance-enhancing substances, drugs, and pharmacological enhancers. Only a minority explicitly specify substances (e.g., anabolic steroids) or distinguish between intentional use and inadvertent exposure, or between physical and cognitive enhancement. Such ambiguity in the definition of doping is characteristic of the field and has been highlighted as a hindering factor in doping behaviour research [135] and communication [18].

## 5. Discussion

The combined bibliometric and narrative analyses reveal a field that is methodologically innovative yet structurally constrained and epistemically fragmented. Three clear issues emerge and are discussed in detail in the sections that follow. First, IEM research remains heavily Western/European in concentration, with only a small core of authors showing sustained engagement. This mirrors broader anti-doping scholarship, where most contributors appear only once in the corpus [113]. Together, these structural patterns depict a research landscape that is productive but fragile and dependent on a small, interconnected community, shaped by disciplinary and gendered pathways, meaning it is vulnerable to epistemic insularity. Second, this study raises questions about what the reported proportions of admitted doping or prohibited substance use can reasonably tell us about doping prevalence in sport. Although estimating national or global prevalence was not the aim, such figures appear across all included outputs, sometimes as primary outcomes and sometimes as methodological by-products. These evidentiary patterns matter because they allow findings to be interpreted in relation to one another and caution against the assumption that “more data” necessarily brings us closer to the truth when it comes to IEMs [136]. Third, the field is characterised by ongoing cycles of method development, refinement, and retrospective re-analysis, either involving new assumptions about the validity of the data (e.g., [34,79]) or adjustments for potential noncompliance (e.g., [65,96]). This continual methodological reworking shapes both the interpretation of existing data and the boundaries of what can be inferred from it.

### 5.1. Geographical Concentration

The pronounced European concentration of published IEM-based doping prevalence studies reflects the contours of the existing literature rather than a bias introduced by this review. Given that the present study is concerned with the development, application, and interpretation of indirect estimation models, this geographic pattern is analytically relevant primarily in terms of ‘where’ and ‘how’ these methods have been deployed to date. Accordingly, the insights generated here are methodologically generalisable but empirically contingent on the contexts in which IEMs have been applied. At the same time, the relative absence of studies from countries with high numbers of confirmed doping cases is a substantive finding in its own right, pointing to structural and institutional gaps in the uptake of IEMs. From a methodological perspective, a more consequential constraint on the field may be the limited range of IEM variants that have been applied to doping prevalence research, despite the availability of additional models [20,137] that, to date, remain under-utilised or unexplored in sports.

Distinct biomedical, behavioural, and sociological framings of doping (as evidenced in the selection of journals, research topics, and thematic areas) correspond to separate intellectual communities, each characterised by their own methodological preferences, publication venues, and linguistic conventions. These invisible colleges shape how doping is studied and communicated, reinforcing parallel, rather than integrated, lines of inquiry. Patterns of authorship concentration and clustering reflect both the strengths and limitations of a specialised community operating at the intersection of behavioural science, statistics, and sport ethics. Given the technical demands of IEMs, such group-specific alignments are not unexpected and resemble developmental trajectories observed in other behavioural domains, such as in the evolution of the Implicit Association Test [138,139,140] and in orthorexia research, where early fragmentation prompted later conceptual consolidation [141,142]. Akin to these examples, the diversity of conceptual and linguistic framings contributes to ongoing ambiguity in how doping is defined, operationalised, and interpreted. Variation in terminology (i.e., how doping is defined and operationalised for data collection) complicates evidence synthesis and cross-study comparison and reflects broader fragmentation across publication outlets [6,50]. Citation patterns further reinforce this dynamic—studies reporting unusually high prevalence estimates tend to attract disproportionate attention, amplifying methodological debates while sometimes sidelining nuance.

### 5.2. Authorship Structure and Implications

Authorship analysis revealed a structurally narrow research community, with only a small group of scholars possessing expertise in both IEM methodology and doping research. Within this small community, the authorship and citation networks show a small number of densely interconnected clusters resembling “invisible colleges” [114], each aligned with particular IEM variants or analytical traditions. These communities facilitate cumulative methodological development but also risk reinforcing established paradigms and limiting cross-fertilisation. Limited dialogue between clusters may entrench methodological divides, slowing conceptual innovation. As Zuccala [143] argues, such invisible colleges persist through the practices of information users (in this case, researchers, policymakers, and critics), whose engagement patterns shape visibility, influence, and impact across the field.

Gender composition adds another layer to the field’s structural dynamics. While women have been comparatively well represented in the broader anti-doping research landscape [113,144], IEM-based prevalence research remains predominantly male-centric. This likely reflects the disciplinary origins of IEM work within quantitative and mathematical traditions, which remain male-dominated globally. Such imbalances may subtly influence the types of questions pursued and the epistemic styles privileged, reinforcing methodological orientations that favour formal modelling and quantification over more contextual or relational approaches to understanding doping behaviour.

This limited pool of experts also raises challenges for expert peer review. Repeated reliance on the same experts risks intellectual insularity, while broadening the reviewer base often brings in specialists who understand either the modelling or the doping context, but not both. These constraints reduce the depth of methodological and contextual evaluation and underscore the need for interdisciplinary collaboration, methodological cross-training, and greater transparency in reviewer expertise.

### 5.3. The Interpretive Scope and Boundaries of ‘Evidence’

The synthesis presented here allows for qualified statements about the relative strength and convergence of IEM-based doping prevalence estimates across models and analytical approaches. By mapping where multiple primary studies align, and where evidence remains sparse or reliant on re-analysis, this review identifies prevalence ranges that are more, or less, strongly supported within the existing literature. In this sense, the analysis clarifies patterns of evidentiary robustness rather than producing a single summary estimate. At the same time, the synthesis does not permit claims about subgroup-specific prevalence, differences by athlete level, or the identification of a definitive or ‘true’ rate of doping, nor does it adjudicate between competing definitions of doping used across studies.

Citation analysis revealed a consistent asymmetry favouring studies with higher or more dramatic prevalence estimates, which tend to attract disproportionate academic and media attention. This pattern suggests that visibility and influence within the field may be shaped as much by the perceived newsworthiness of findings as by methodological innovation or quality. While such attention can raise awareness of doping as a social issue, it risks overshadowing more nuanced or conservative studies that may offer greater validity. Moreover, the probabilistic nature of IEM-derived estimates makes them vulnerable to misinterpretation by audiences unfamiliar with indirect estimation principles. Without appropriate context, these figures may be misconstrued as direct evidence of doping rates rather than statistical inferences. Authors, reviewers, and editors therefore share responsibility for clear and transparent communication—providing interpretive guidance, confidence intervals, and explicit caveats to prevent sensationalism and misuse of complex quantitative data.

Interpretation of IEM-derived prevalence estimates must be situated within the broader sociocultural context of elite sport. As noted by the reviewers, historical accounts of state-sponsored doping [145,146,147,148], normalisation narratives (e.g., claims that “everyone does it”), and what has been described as a “code of silence” [149,150,151,152] highlight the possibility of systematic denial that may constrain disclosure even under indirect questioning. As with all self-report–based methods, IEMs rely on respondent engagement, instruction compliance, and willingness to disclose sensitive behaviours; consequently, their estimates should be understood as reflecting self-admitted, rather than objectively verified, doping prevalence. While IEMs are specifically designed to mitigate social desirability bias and underreporting, they cannot fully overcome structural or cultural forces that promote denial or normalise concealment. These contextual dynamics likely impose an upper bound on what any survey-based approach (direct or indirect) can capture and should be considered when interpreting prevalence estimates.

A notable gap across the corpus is the absence of qualitative or mixed-method work exploring how respondents understand and engage with IEM surveys. Existing evidence suggests that comprehension, trust, and emotional responses influence data quality and may contribute to noncompliance [29,30,31,153]. Without insight into these processes, refinements in statistical modelling risk outpacing understanding of respondent behaviour [154,155,156]. Incorporating qualitative methods such as cognitive interviews or think-aloud protocols could help distinguish between true concealment and methodological artefacts and provide a behavioural foundation for future model development. These limitations also intersect with the field’s Eurocentric orientation. Most IEM-based doping prevalence studies have been designed and interpreted within Western European contexts, raising questions about cultural transferability. Assumptions about privacy, probabilistic reasoning, and institutional trust may not hold globally, making cross-cultural and qualitative validation essential for ensuring conceptual, ethical, and measurement robustness.

### 5.4. Duplicate Publications and Re-Analyses

Duplicate publications and secondary data re-analyses pose distinct challenges for evidence synthesis and bibliometric evaluation, particularly in method-intensive research domains. Often justified on grounds of audience reach, language accessibility, or disciplinary targeting, duplicate publications can complicate systematic reviews by increasing the risk of inadvertent data duplication and can distort bibliometric indicators by inflating publication and citation counts [157]. These effects are especially salient in specialised fields with small expert communities and limited primary datasets, where the same empirical material may legitimately circulate across multiple outlets.

Secondary data analysis and re-analyses, by contrast, are a normal [158] and often scientifically necessary component of cumulative knowledge building, particularly as methodological assumptions evolve, or new analytical tools become available. In IEM-based doping prevalence research, such re-analyses typically arise from refined assumptions regarding survey instruction noncompliance, response validity, or model specification. These methodological iterations can enhance model robustness and theoretical clarity, but they also introduce interpretive complexity when multiple, equally plausible prevalence estimates are derived from the same dataset, each contingent on different behavioural or statistical assumptions. Although scientifically valuable, secondary analyses of the same dataset under different assumptions create practical challenges for meta-analysis and evidence synthesis. Researchers conducting quantitative syntheses must therefore either (i) predefine objective decision rules to select a single estimate per dataset or (ii) include multiple estimates and explicitly account for their statistical dependence arising from shared underlying data.

Concerns about redundancy often emerge not from re-analysis itself, but from ambiguity regarding analytic intent, data provenance, and the distinctiveness of contributions. In this sense, re-analysis can create a grey zone in which legitimate methodological refinement may, if insufficiently documented, be difficult to distinguish from publication practices that raise ethical or epistemic concerns [159]. Without explicit disclosure of dataset reuse, analytical rationale, and the nature of departures from original analyses, repeated publications may be perceived—rightly or wrongly—as instances of selective reporting, p-hacking, undisclosed post hoc hypothesising (i.e., HARKing, RHARKing, CHARKing), or salami slicing [160,161,162], even in the absence of questionable research practice (QRP) intent. In this context, salami slicing should be understood not as a rule defined by numeric thresholds or dataset reuse *per se*, but as a risk of misclassification as such by editors, reviewers, or peers that arises when analytical boundaries, research questions, or justificatory rationales are insufficiently transparent.

### 5.5. Practical Implications

These findings have several implications for researchers, practitioners, and journal editors operating at the intersection of sports science, behavioural research, and anti-doping policy. For researchers, the results underscore the importance of transparent reporting and reflexivity when applying IEMs. Clear documentation of data provenance, analytical assumptions, and the rationale for re-analysis should become standard practice to reduce duplication bias and enhance interpretive clarity. Cross-disciplinary training integrating behavioural science, psychometrics, and sport ethics may further strengthen methodological and contextual competence.

For practitioners and anti-doping organisations, a nuanced understanding of IEM-derived prevalence estimates is essential. As these estimates are probabilistic rather than diagnostic, they are best suited to informing population-level strategy rather than individual-level judgement. Training and communication materials should therefore emphasise the interpretive limits of IEM outputs and situate them within broader evidence frameworks, including testing statistics, education programme indicators, and sociocultural data.

For journal editors and reviewers, diversifying the peer-review process is critical. Engaging reviewers with complementary methodological and applied expertise (rather than relying on a narrow group of specialists) can mitigate intellectual clustering and promote balanced evaluation. Editors may also consider requiring explicit statements on data reuse, analytical transparency, and open-science compliance. Across all stakeholder groups, greater attention to cultural and linguistic sensitivity in the design, analysis, and dissemination of IEM-based research is essential to enhance trust, data quality, and the ethical integrity of future doping prevalence studies.

Clearer reporting standards for re-analysis and secondary data analysis are essential. Explicit documentation of data provenance, analytical degrees of freedom, pre-registration (or amendment histories), and adherence to open-science principles can help distinguish genuine methodological advancement from ethically problematic redundancy [163,164]. In complex modelling fields such as IEM-based prevalence estimation, such transparency is not merely an ethical safeguard but a prerequisite for interpretive coherence, cumulative validity, and sustained trust among scientific, policy, and practitioner audiences.

At the same time, a growing body of work promotes secondary data analysis and re-analysis as sustainable, efficient, and resource-effective research practices (e.g., [164,165,166,167]). Such approaches are commonly justified on grounds of reducing financial and environmental costs, maximising the utility of existing datasets, and enabling sustainability-oriented research infrastructures (for example, open-science ecosystems [168] and FAIR data principles [169]). The situational resemblance between promoting open science and re-analysis [170], the trajectory of debate around implicit measures such as the implicit association test [139,140], and the estimation of admitted sensitive transgressions in IEMs are noteworthy. In each case, researchers can quantify and statistically analyse patterns in data that are assumed to reflect how people respond to a cognitively demanding task built on theoretical assumptions about behaviour (i.e., how participants ‘solve’ that test or survey task). Yet, the underlying behavioural and cognitive processes are not directly observable. They may be shaped by confounds (e.g., attention, motivation, and response strategies) and can distort the estimation as well as the interpretation of the results. Therefore, progress with IEM development and refinement should rely on iterative empirical studies, replication, and re-analysis, through which evidence accumulates and understanding develops incrementally.

Against this backdrop, IEMs should not be treated as simple ‘plug-and-play’ tools. As the results of this review illustrate, the field has moved from straightforward application toward questioning, critically appraising, and stress-testing key assumptions underpinning IEM-based estimation, paralleling how implicit measures evolved from widespread adoption to sustained critique, refinement, and reassessment of what constructs they actually capture [140,170]. Extending this parallel, IEM research has become increasingly attentive to behavioural dynamics that shape motivated truthfulness, self-protective responding, trust and understanding, and inattentive or random responding, thus affecting the fundamental statistical assumptions [76,79].

### 5.6. Study Limitations

This study has limitations that need to be acknowledged. Bibliometric analyses were possible only for outputs indexed in Web of Science and Scopus, introducing potential database selection bias toward English-language publications and higher-impact journals and excluding studies found in regional or non-indexed outlets. Likewise, the narrative component focused on English-language publications, limiting interpretive depth for studies available only in other languages. In evaluating evidentiary strength, estimates were not disaggregated by athlete sport involvement level or by definition of doping, but rather, they focused on IEMs and whether the analysis assumed and accounted for noncompliance in some form of secondary analysis. Readers needing detailed quantitative synthesis should consult Sagoe et al. [50].

The authors of this review contributed to some of the analysed outputs, presenting a challenge to complete impartiality. This was mitigated through objective bibliometric procedures, transparent inclusion criteria, and involvement of bibliometric experts without prior publications in the doping field. Nonetheless, our narrative interpretations inevitably reflect our own epistemic orientations. We therefore foreground positionality as part of reflexive and mixed-method scholarship. The authorship team’s disciplinary balance, gender diversity, and varied involvement in anti-doping contribute to epistemic breadth rather than bias.

### 5.7. Future Directions

Future work should expand bibliometric coverage beyond Web of Science and Scopus to include regional and language-specific databases, reducing indexation bias and offering a more global picture of IEM use. Multilingual narrative analyses would further illuminate how conceptualisations and reporting practices vary across cultural contexts. We feel that this is an important consideration given that the framing of sensitive behaviours like doping is shaped by cultural norms and moral discourses.

Greater attention to participant experience with IEMs is also needed. Although IEMs are designed to protect anonymity, their validity depends on respondents’ comprehension, trust, and motivation. Evidence from sensitive survey research highlights the role of misunderstanding, self-protection, or disengagement in driving noncompliance. Cognitive interviewing, think-aloud protocols, and cross-cultural piloting would clarify these behavioural processes and improve model robustness. Understanding and modelling instruction noncompliance remains a priority, given its central role in biassing prevalence estimates. Future studies should triangulate theoretical models of noncompliance with empirical behavioural data—via experiments, response time analysis, or behavioural tracking—to refine correction procedures and strengthen interpretive validity. Over the next decade, priorities should therefore centre on refinement and reassessment (potentially in combination with a complementary method) to better characterise the behavioural and cognitive processes behind truthful answers, false positives, and false negatives. Ultimately, the field requires robust and defensible ways of handling compliance and noncompliance if prevalence claims about doping are to rest on IEM-based estimates.

Cross-community collaboration remains essential for mitigating the intellectual insularity sustained by methodological and disciplinary “invisible colleges’’. Interdisciplinary research bridging statistical modelling with sports science, behavioural science, and ethics would broaden interpretive perspectives. Joint authorship, shared data repositories, open methodological documentation, and interdisciplinary symposia could promote methodological learning and reduce fragmentation. The field would also benefit from specialised methodological guidelines for evidence synthesis in IEM-based doping research. Current systematic-review frameworks are not well equipped to handle duplicated outputs, re-analyses, and cross-model heterogeneity. Developing consensus-based standards analogous to PRISMA extensions tailored to complex modelling and IEMs would enhance transparency, reduce duplication bias, and improve comparability across studies.

## 6. Conclusions

This study complements existing doping prevalence estimates and their systematic and meta-analytic synthesis [50] by situating IEMs within their intellectual, social, and methodological ecosystems. Understanding these dynamics helps place IEM-based prevalence estimates into policy, practical, and research contexts, and cautions against the ‘higher must be more truthful’ heuristic, selective citation, and overinterpretation. Over time, IEM-based doping prevalence research has evolved from early prevalence reporting toward greater methodological reflexivity and specialisation. Despite increased internal coherence and visibility, the field remains constrained by Eurocentrism, intellectual clustering, and uneven interpretive standards. Importantly, the identification of Eurocentrism, intellectual clustering, and fragmentation in this review is intended as a diagnostic contribution rather than a corrective intervention. Addressing these structural imbalances would require new primary research, broader geographic investment, and institutional diversification, which are, by design, beyond the remit of a bibliometric and interpretive analysis.

To date, IEM-based doping prevalence research has focused almost exclusively on either statistical method development or straightforward application to generate prevalence estimates, with a notable gap in studies examining how athletes perceive, experience, and respond to sensitive doping questions within IEM survey environments. Attention to this gap is critical for understanding survey instruction noncompliance and for informing robust post-data-collection adjustment strategies. Future priorities include cultural adaptation of IEM instruments, qualitative investigation of behavioural dynamics, triangulation with empirical data, and the development of method-specific standards for synthesising IEM-derived prevalence estimates, including adjustments for survey instruction noncompliance. Addressing these limitations is essential to ensure that prevalence estimates are not only statistically robust but also ethically sound, culturally grounded, and fit for informing anti-doping policy and governance. Given the methodological complexity of IEMs, new users are strongly encouraged to collaborate with experienced experts. Policymakers and practitioners should also pay close attention to the intended purpose of prevalence studies, distinguish method development from prevalence estimation, and exercise considerable caution when interpreting estimates derived from studies focusing on methodological improvement and validation.

## Figures and Tables

**Table 2:** Evidence strength mapping across the IEM-based prevalence studies.

	0–5		6–10		11–15		16–20		21–25		26–30		31–35		36–40		41–45		46–50		51–55		56–60		61–65		66–70		71–75		76–80		80+	
FR	14	6	5	1	2				2		1																							
UQM	3		9		5	1	1			1							1						2											
CM	1			1	1	3	3	3	2	1				1	1	1							1								1			
SSC		7	1	2	1	4	1			4	1					2																		
Kuk’s	6	5	3	5	1																													

Blue: unadjusted; orange: adjusted for noncompliance (re-analysis with a set of assumptions about noncompliance) and subgroup analyses. Shading indicates frequency of studies in that particular cell (darker shades denote higher frequency counts). Limited to peer-reviewed outputs (journal articles and book chapters). FR: forced response, UQM: Unrelated Question Model, CM: Crosswise Model, SSC: Single Sample Count.

## Data Availability

No new data were created or analysed in this study. Data sharing is not applicable to this article.

## Supplementary Materials

The following supporting information can be downloaded at: https://www.mdpi.com/article/10.3390/sports14060229/s1, Table S1: Distribution of the included scientific journal articles (*k* = 33) by journals; Table S2: Distribution of the outputs indexed in WoS (*k* = 26) by research topics (in all collections); Table S3: Scientific impact assessment; Table S4: Evidentiary summary table with references; Table S5: List of authors involved in IEM application to doping prevalence estimation.

## Abbreviations

The following abbreviations are used in this manuscript:

CHARKing	Cherry-picking significant results
CW	Crosswise Model
FR	Forced-response model
FWCI	Field-Weighted Citation Index
IEM	Indirect estimation model
RHARKing	Retrieving hypotheses from post hoc literature searches
Salami slicing	Fragmenting one study across multiple research outputs
SHARKing	Suppressing unsupported a priori hypotheses
SSC	Single Sample Count Model
UQM	Unrelated Question Model

## Appendix A

**Table A1:** Comparison of IEMs used in doping prevalence studies focusing on respondent experience and face validity.

Model Family/Example	How the Question Is Experienced by Respondents	How Respondents’ Answers Are Protected	Face Validity: How It Feels like a Doping Survey	Forced Affirmative Response and Its Implications	Detecting Survey-Instruction Noncompliance
Combined-response models (e.g., Crosswise Model)	Respondents answer the doping question together with a neutral question, reporting only whether the answers match	Individual answers are concealed by combining responses to the sensitive question and other unrelated non-sensitive questions	High: all respondents perceive that they are answering the doping question	No forced “yes”; protection relies on ambiguity of combined answers	Requires two parallel versions and a randomly split sample
Randomised-response models (e.g., forced response, Kuk’s design)	Respondents follow instructions that sometimes require answering the doping question and sometimes require a preset answer	Protection is achieved because, for the researcher, forced and genuine “yes” answers to the sensitive question are indistinguishable	Moderate: not all respondents feel they meaningfully answered the doping question	Yes: some respondents must say “yes” regardless of behaviour, which may reduce comfort and increase noncompliance	Requires two parallel versions and a randomly split sample
Question-substitution models (e.g., Unrelated Question Model)	Respondents answer either the doping question or a harmless question, determined by chance	Researchers cannot identify who answered which question (the sensitive or the unrelated question)	Moderate: only part of the sample directly answers the doping question	No forced “yes”; protection depends on question substitution	Requires two parallel versions and a randomly split sample
Count-based models (e.g., Single Sample Count)	Respondents report how many statements apply, without specifying which ones	Individual responses remain fully concealed through aggregation of the responses, of which the sensitive question is only one of many	High: respondents feel included, but the doping question is indirect	No forced “yes”; protection comes from lack of item-level disclosure	Does not require two parallel versions if the non-sensitive questions are set to known prevalences (e.g., distribution of birth dates)
