# Supplementary material for: Charting the Scientific Landscape of Indirect Estimation Models in Doping Prevalence Research: A Bibliometric Analysis with Narrative Appraisal
Source: Sports (Basel). 2026 Jun 3;14(6):229. doi: 10.3390/sports14060229 (PMC13306287; doi:10.3390/sports14060229)
Supplement: Supplementary file 1 [file sports-14-00229-s001.zip › Sports IEM review Table S1.pdf]

Supplementary Table S1. Distribution of the included scientific journal articles (k = 33) by journals. Sport focused journals are highlighted in grey.

Journal	Best JIF quartile	Frequency
1. *PLOS One*	Q1	4
2. *Sports Medicine-Open*	Q1	4
3. *Performance Enhancement and Health*	Q2	3
4. *Addiction*	Q1	1
5. *Addiction and Health*	Not ranked	1
6. *Behavior Research Method*	Q1	1
7. *Drug and Alcohol Dependence*	Q1	1
8. *Drug Testing and Analysis*	Q2	1
9. *Epidemiology*	Q1	1
10. *European Journal for Sport and Society*	Q1	1
11. *Frontiers in Psychology*	Q2	1
12. *Frontiers in Sports and Active Living*	Q1	1
13. *International Review for the Sociology of Sport*	Q1	1
14. *Journal for ReAttach Therapy and Developmental Diversities*	Q4	1
15. *Panopticon: Journal of Criminal Law, Criminology and Criminal Justice*	Not ranked	1
16. *Journal of Risk and Financial Management*	Q2	1
17. *Journal of Sports Sciences*	Q1	1
18. *Journal of Survey Statistics and Methodology*	Q1	1
19. *Journal of the Royal Statistical Society: Series A*	Q1	1
20. *Medicine and Science in Sport and Exercise*	Q1	1
21. *Psychology of Sport and Exercise*	Q1	1
22. *Psychological Methods*	Q1	1
23. *Psychometrika*	Q1	1
24. *Scandinavian Journal of Medicine & Science in Sports*	Q1	1
25. *Scientific Journal of Sport and Performance*	Not ranked	1
26. *Sports Medicine*	Q1	1
