# Supplementary material for: Charting the Scientific Landscape of Indirect Estimation Models in Doping Prevalence Research: A Bibliometric Analysis with Narrative Appraisal
Source: Sports (Basel). 2026 Jun 3;14(6):229. doi: 10.3390/sports14060229 (PMC13306287; doi:10.3390/sports14060229)
Supplement: Supplementary file 1 [file sports-14-00229-s001.zip › Sports IEM review Table S2.pdf]

Supplementary Table S2. Distribution of the outputs indexed in WoS (*k* = 26) by research topics (in all collections)
One output is classed in multiple topics.

**Articles (by first author)**	**Behavioural Sciences**	**Biochemistry Molecular Biology**	**Business Economics**	**Chemistry**	**Criminology Penology**	**Demography**	**Education, Educational Research**	**General Internal Medicine**	**Geriatrics Gerontology**	**Health Care Sciences Services**	**Infectious Diseases**	**Information Science Library Sciences**	**Legal Medicine**	**Mathematics**	**Mathematical Computational Biology**	**Mathematical Methods in Social**	**Medical Ethics**	**Neurosciences Neurology**	**Orthopedics**	**Pediatrics**	**Pharmacology Pharmacy**	**Psychiatry**	**Psychology**	**Public Environmental Occupational**	**Rehabilitation**	**Research Experimental Medicine**	**Respiratory System**	**Science Technology Other**	**Sociology**	**Social Sciences Other**	**Sport Sciences**	**Substance Abuse**	**Toxicology**
[62] Boardley et al. (2019)	1	0	0	0	0	0	1	1	0	0	0	0	0	0	1	0	1	0	1	1	0	0	1	0	1	0	0	0	1	0	1	0	0
[64] Christiansen et al. (2023)	0	0	0	0	0	0	0	0	0	0	0	0	0	0	0	0	0	0	1	0	0	0	0	0	1	0	0	0	0	0	1	0	0
[65] Cruyff et al. (2024)	0	0	1	0	0	0	0	0	0	0	0	0	0	1	0	1	0	0	0	0	0	0	0	0	0	0	0	0	0	0	0	0	0
[66] Dietz et al. (2013)	0	0	0	0	0	1	0	1	1	0	0	0	0	0	0	0	0	0	0	1	1	1	0	0	0	0	0	1	1	0	1	1	0
[67] Dietz et al. (2016)	1	0	0	0	0	1	0	1	0	0	0	0	0	1	0	0	0	0	0	0	1	0	0	0	0	0	0	0	1	1	1	0	1
[71] Franke et al. (2017)	1	0	0	0	0	0	0	0	0	1	0	0	0	0	0	0	0	0	0	0	1	1	1	1	0	1	0	0	0	1	0	0	0
[72] Frenger et al. (2016)	0	0	0	0	0	0	1	1	0	0	0	0	0	0	0	0	0	0	0	0	1	1	0	0	0	0	0	1	1	1	1	0	1
[73] Heller et al. (2020)	1	0	0	0	0	0	0	0	0	0	0	0	0	0	0	0	0	0	0	0	0	0	1	0	0	0	0	0	0	0	0	0	0
[75] Hilkens et al. (2021)	0	0	0	0	0	0	0	0	0	0	0	0	0	0	0	0	0	0	1	0	0	0	0	0	1	0	0	0	0	0	1	0	0
[76] James et al. (2013)	1	0	0	0	0	0	0	0	0	0	0	0	0	0	0	0	0	0	0	0	0	0	1	0	0	0	0	0	0	1	1	0	0
[79] Petróczi et al. (2022)	0	0	0	0	0	0	0	0	0	0	0	0	0	0	0	0	0	0	0	0	0	0	0	0	0	0	0	0	0	0	1	0	0
[81] Pitsch (2022)	0	0	1	0	0	0	0	0	0	0	0	0	0	0	0	0	0	0	0	0	0	0	0	0	0	0	0	0	0	0	0	0	0
[83] Pitsch and Emrich (2012)	0	0	0	0	0	0	0	0	0	0	0	1	0	1	0	0	0	0	0	0	0	0	1	0	0	0	0	0	1	1	1	0	0
[91] Reiber et al. (2022)	1	0	0	0	0	0	0	0	0	0	0	0	0	0	0	0	0	0	0	0	0	0	1	0	0	0	0	0	0	0	0	0	0
[93] Sayed et al. (2022)	0	0	0	0	0	0	0	1	0	0	1	0	0	0	1	0	0	0	0	0	0	1	0	1	0	0	1	1	1	0	1	0	1
[97] Schröter et al. (2016)	1	0	0	0	0	1	0	1	1	0	0	0	0	1	1	0	0	0	0	0	0	0	0	0	0	0	0	1	1	0	1	0	0
[99] Seifarth et al. (2019)	0	0	0	0	0	0	0	0	0	0	0	0	0	0	0	0	0	0	0	0	0	0	0	0	0	0	0	0	0	0	1	0	0

[100] Simon et al. (2006)	0	0	0	0	0	1	0	0	0	1	0	0	0	1	0	0	0	0	0	0	1	1	1	0	0	0	0	0	1	0	1	1	1
[102] Striegel et al. (2010)	1	0	0	0	0	1	0	1	0	0	0	0	0	0	0	0	0	1	0	1	0	1	0	0	0	0	0	1	1	1	1	1	1
[104] Stubbe et al. (2013)	0	1	0	1	0	0	0	0	0	1	0	0	0	0	0	0	0	0	0	0	1	0	0	0	0	0	0	0	0	0	0	0	0
[105] Ulrich et al. (2018)	0	0	0	0	0	1	0	0	0	0	0	0	0	0	0	0	0	0	1	9	9	9	9	9	1	0	0	0	1	1	1	1	1
[34] Ulrich et al. (2023)	0	0	0	0	0	0	0	0	0	0	0	0	0	0	0	0	0	0	1	0	0	0	0	0	1	0	0	0	0	0	1	0	0
[94] Sayed et al. (2024a)	1	0	0	0	1	0	0	0	0	0	0	0	1	1	1	0	0	0	0	0	0	0	1	0	0	0	0	0	0	0	0	0	0
[95] Sayed et al. (2024b)	1	0	0	0	0	1	0	0	0	0	0	0	0	1	1	0	0	0	0	0	1	0	1	0	0	0	0	0	0	0	0	0	0
[98] Schu & Haller (2026)	0	0	0	0	0	0	0	0	0	0	0	0	0	0	0	0	0	0	0	0	0	0	1	0	0	0	0	0	0	1	1	1	0
[82] Pitsch & Christiansen (2026)	0	0	0	0	0	0	0	0	0	0	0	0	0	0	0	0	0	0	0	0	0	0	1	0	0	0	0	0	0	1	1	1	0
[96] Sayed et al. (2026)	0	0	0	0	0	0	0	0	0	0	0	0	0	1	0	1	0	0	0	0	0	0	0	0	0	0	0	0	0	0	0	0	0
**Total**	**10**	**1**	**2**	**1**	**1**	**7**	**2**	**7**	**2**	**3**	**1**	**1**	**1**	**8**	**5**	**2**	**1**	**1**	**5**	**12**	**16**	**15**	**20**	**11**	**5**	**1**	**1**	**5**	**10**	**9**	**18**	**6**	**6**
