# Supplementary material for: Charting the Scientific Landscape of Indirect Estimation Models in Doping Prevalence Research: A Bibliometric Analysis with Narrative Appraisal
Source: Sports (Basel). 2026 Jun 3;14(6):229. doi: 10.3390/sports14060229 (PMC13306287; doi:10.3390/sports14060229)
Supplement: Supplementary file 1 [file sports-14-00229-s001.zip › Sports IEM review Table S3.pdf]

Supplementary Table S3: Scientific Impact assessment

**Outputs**	**Web of Science** as of 26/01.2026		**Scopus** as of 01/02/2026		
	**Total citation**	**Avg. citation per year**	**FWCI**	**SciVal Topic Prominence**	**SciVal Topic**
[62] Boardley et al. (2019)	17	2.13	0.86	82.932	Doping Policies and Athlete Integrity in Sports
[64] Christiansen et al. (2023)*	23	5.75	4.09	81.814	Randomized Response Techniques for Sensitive Surveys
[65] Cruyff et al. (2023)	0	0	0	81.814	Randomized Response Techniques for Sensitive Surveys
[66] Dietz et al. (2013)	58	4.14	1.03	85.100	Prescription Drug Misuse and Cognitive Enhancement
[67] Dietz et al. (2016)	21	1.91	1.73	87.103	Nutritional Supplements and Athlete Performance Insights
[69] Elbe and Pitsch (2018)	38**		2.24	82.932	Doping Policies and Athlete Integrity in Sports
[71] Franke et al. (2017)	11	1.1	0.68	85.100	Prescription Drug Misuse and Cognitive Enhancement
[72] Frenger et al. (2016)	19	1.73	0.52	82.932	Doping Policies and Athlete Integrity in Sports
[73] Heller et al. (2020)	6	0.86	0.33	82.932	Doping Policies and Athlete Integrity in Sports
[75] Hilkens et al. (2021)	66	11	6.34	94.116	Health Risks of Anabolic Steroid Use
[76] James et al. (2013)	19	1.36	1.53	81.814	Randomized Response Techniques for Sensitive Surveys
[79] Petróczi et al. (2022)	14	2.8	1.54	81.814	Randomized Response Techniques for Sensitive Surveys
[85] Pitsch et al. (2007)	91**	-	0.58	82.932	Doping Policies and Athlete Integrity in Sports
[81] Pitsch (2022)	5	1	0.41	82.932	Doping Policies and Athlete Integrity in Sports
[83] Pitsch and Emrich (2012)	90	6	7.75	82.932	Doping Policies and Athlete Integrity in Sports
[82] Pitsch & Christiansen (2026)*	0	0	0	82.932	Doping Policies and Athlete Integrity in Sports
[91] Reiber et al. (2022)	6	1.2	0.45	81.814	Randomized Response Techniques for Sensitive Surveys
[92] Robach et al. (2024)	5**	-	1.85	76.894	Erythropoietin Doping and Detection Methods
[93] Sayed et al. (2022)	5	1	0.29	81.814	Randomized Response Techniques for Sensitive Surveys
[94] Sayed et al. (2024a)*	0	0	0	81.814	Randomized Response Techniques for Sensitive Surveys
[95] Sayed et al. (2024b)*	3	0.75	0.95	81.814	Randomized Response Techniques for Sensitive Surveys
[96] Sayed et al. (2026)*	0	-	0	81.814	Randomized Response Techniques for Sensitive Surveys
[97] Schröter et al. (2016)	27	2.45	0.64	81.814	Randomized Response Techniques for Sensitive Surveys
[98] Schu & Haller (2026)	1	1	1.5	85.100	Prescription Drug Misuse and Cognitive Enhancement
[99] Seifarth et al. (2019)	11	1.38	0.62	82.932	Doping Policies and Athlete Integrity in Sports

[100] Simon et al. (2006)	89	4.24	1.18	94.116	Health Risks of Anabolic Steroid Use
[102] Striegel et al. (2010)	111	6.53	3.54	82.932	Doping Policies and Athlete Integrity in Sports
[104] Stubbe et al. (2013)	55	4.23	1.7	94.116	Health Risks of Anabolic Steroid Use
[105] Ulrich et al. (2018)	144	16	10.38	82.932	Doping Policies and Athlete Integrity in Sports
[34] Ulrich et al. (2023)*	2	0.5	0.39	81.814	Randomized Response Techniques for Sensitive Surveys
Mean			1.771		
SD			2.429		
median			0.905		
Trimmed mean (80%)			1.526		

* denotes re-analysis of previously reported data; ** taken from Scopus

Definitions:

Field-Weighted Citation Impact (FWCI) is the ratio of citations received relative to the expected world average for the subject field, publication type, and publication year. Field-Weighted Citation Impact takes into account the differences in research behaviour across disciplines. With FWCI = 1, the output performs as expected for the global average. Conversely, FWCI > 1 means that the output is more cited than expected, and FWCI < 1 means that the output is cited less than expected in its respective field.

Topics are multidisciplinary collection of documents with a common intellectual interest in SciVal that represent fields of research, based on clustering the citation network of 95% of Scopus content (all documents published from 1996). Each Topic is clustered within SciVal based upon direct citation analysis using document reference lists with hard clustering (i.e., a document can belong to only one topic). Topics dynamic, as newly published documents are indexed, they change and are likely to grow over time.

Topic Prominence is a composite index of three metrics which indicate the momentum of the topic within a field of study and ranks the topic by prominence of these citation patterns. The three metrics are: (1) Citation count in year n to papers published in n and n-1; and (2) Scopus view count in year n to papers published in n and n-1; and (3) Average Journal CiteScore for year n.

References:

Scopus SciVal: https://service.elsevier.com/app/answers/detail/a_id/27947/supporthub/scopus/kw/topics/
https://service.elsevier.com/app/answers/detail/a_id/14894/supporthub/scopus/kw/FWCI/
