# Supplementary material for: Charting the Scientific Landscape of Indirect Estimation Models in Doping Prevalence Research: A Bibliometric Analysis with Narrative Appraisal
Source: Sports (Basel). 2026 Jun 3;14(6):229. doi: 10.3390/sports14060229 (PMC13306287; doi:10.3390/sports14060229)
Supplement: Supplementary file 1 [file sports-14-00229-s001.zip › Sports IEM review Table S4.pdf]

Supplementary Table S4: Evidentiary summary table with references

Fractional values are assigned to bins using upward rounding; thus, any estimate exceeding a bin boundary (e.g., 5.1%) is categorised in the next 5% interval (e.g., 6–10%).

Bins (%)	Unadjusted estimates	Adjusted estimates/Secondary analysis
**0–5**	Christiansen et al. (2023)[64]; Elbe and Pitsch (2018)[69]; Frenger et al. (2016)[72]; Hilkens et al. (2021)[75]; Pitsch (2018)[80]; Pitsch (2022)[81]; Robach et al. (2024)[92]; Sayed et al. (2022)[93]; Sayed et al. (2024a)[94]; Sayed et al. (2024b)[95]; Schröter et al. (2016)[97]	Petróczi et al. (2022)[79]; Pitsch & Christiansen (2026)[82]; Sayed et al. (2024a)[94]; Sayed et al. (2024b)[95]
**6–10**	Fincoeur & Pitsch (2017)[70]; Franke et al. (2017)[71]; Hilkens et al. (2021)[758]; Nilaweera et al. (2020)[78]; Petróczi et al. (2022)[79] Pitsch (2018)[80]; Pitsch (2022)[81]; Reiber et al. (2022)[91]; Sayed et al. (2024a)[94]; Schröter et al. (2016)[97]; Schu & Haller (2025)[98]; Seifarth et al. (2019)[99]; Striegel et al. (2010)[102]; Stubbe et al. (2014)[52]	Petróczi et al. (2022)[79]; Pitsch & Christiansen (2026)[82]; Sayed et al. (2024a)[94]; Sayed et al. (2024b)[95]; Sayed et al. (2026)[96]
**11–15**	Balk et al. (2023)[61]; Boardley et al. (2019)[62] Dietz et al. (2013)[66]; Heller et al. (2020)[73]; Petróczi et al. (2022)[79]; Pitsch & Emrich (2012)[83]; Pitsch et al. (2007)[85]; Sayed et al. (2022)[93]; Schröter et al. (2016)[97]; Simon et al. (2006)[100]	Cruyff et al. (2024)[65]; Dietz et al. (2016)[67] Petróczi et al. (2022)[79]; Sayed et al. (2026)[96]
**16–20**	Cruyff et al. (2024)[65]; Dietz et al. (2013)[66]; James et al. (2013)[76]; Sayed et al. (2022)[93]	Cruyff et al. (2024)658]; Sayed et al. (2026)[96]
**21–25**	Cruyff et al. (2024)[65]; Pitsch et al. (2007)[38]	Cruyff et al. (2024)[65]; Dietz et al. (2016)[67]; Petróczi et al. (2022)[79]
**26–30**	Cruyff et al. (2024)[65]; Pitsch et al. (2007)[85]	
**31–35**		Sayed et al. (2026)[96]
**36–40**	Cruyff et al. (2024)[65]	Cruyff et al. (2024)[65]; Ulrich et al. (2023)[34]
**41–45**	Ulrich et al. (2018)[105]*	
**56–60**	James et al. (2013)[76]*; Nakhaee et al. (2013)[77]; Ulrich et al. (2018)[105]*	
**76–80**	Abdulrazzaq and Tareq (2023)[58]	

* Using the same UQM variant where respondents can choose the randomisation (person's birthday)
