# Supplementary material for: Charting the Scientific Landscape of Indirect Estimation Models in Doping Prevalence Research: A Bibliometric Analysis with Narrative Appraisal
Source: Sports (Basel). 2026 Jun 3;14(6):229. doi: 10.3390/sports14060229 (PMC13306287; doi:10.3390/sports14060229)
Supplement: Supplementary file 1 [file sports-14-00229-s001.zip › Sports IEM review Table S5.pdf]

Supplementary Table S5. List of authors involved in IEM application to doping prevalence estimation.

Author	2002	2003	2004	2005	2006	2007	2008	2009	2010	2011	2012	2013	2014	2015	2016	2017	2018	2019	2020	2021	2022	2023	2024	2025	2026	Total
Abdulrazzaq, Zeyad																						1				1
Aust, Fabian					1																					1
Backhouse, Susan															1											1
Balk, Lisanne																				1		1				2
Balló, Harald																1										1
Beggs, Clive															1											1
Benjamins, Jeroen																				1						1
Boardley, Ian																		1								1
Botre, Franceso																							1			1
Bouzat, Pierre																							1			1
Breuer, Christoph												1														1
Buisson, Corinne																							1			1
Chirico, Andrea																						1				1
Chorus, Astrid													1													1
Christiansen, Ask																						1			1	2
Cléret, Lea																	1					1				2
Comstock, Dawn																	1					1				2
Cruyff, Maarten																				1	1	1	4		1	8
Dalaker, Robert												1			1											2
De Hon, Olivier													1	1							1	1				4
De Seigneux, Sophie																							1			1
Dietz, Pavel					1							1			2	1		1	1							7
Disch, Alexander																		1								1
Dopheide, Marit																				1		1				2
Duiven, Erik														1								1				2
Elbe, Anna-Marie																	1									1

Emrich, Eike				1		1		2			1	1			1											7
Engelhardt, Martin																		1								1
Evers, Catharine																				1						1
Fincoeur, Bertrand																1										1
Frank, Laurence													1													1
Franke, Andreas												1				1										2
Frenger, Monica												1			1							1				3
Garribba, Flaminia																							1			1
Gebert, Angela										1																1
Giardini, Guido																							1			1
Gleaves, John																					1					1
Gucciardi, Daniel																		1								1
Haller, Niels																									1	1
Hallmann, Kristin												1														1
Harris, Tyler																		1								1
Heller, Sebastian																			1							1
Heyes, Andrew																					1					1
Hilkens, Luuk																				1						1
James, Ricky												1														1
Kamber, Matthias										1																1
Kanayama, Gen																	1					1				2
Klein, Marcus				1		1																				2
Lamprecht, Markus										1																1
Lefevre, Brice																							1			1
Letzel, Stephan															1											1
Lieb, Klaus												1				1										2
Maats, Peter								2																		2
Mazzarino, M																							1			1
McKenna, Jim															1											1
Mechin, Natalie																							1			1

Musch, Joachim	1																									1
Nadishani, Upuli																			1							1
Nakhaee, Mohammad												1														1
Nakhaee, Nouzar												1														1
Naughton, Declan												1														1
Nepusz, Tamas												1					1									2
Nilaweera, Akhila																			1							1
Nipunya, Geeth																			1							1
Ntoumanis, Nikos																		1								1
Nunn, Richard															1											1
Pakravan, Faezeh												1														1
Petróczi, Andrea												1			1		1				2	1	1		1	8
Pitsch, Werner				1		1		2			1	1			1	1	2				1	1			1	13
Plessner, H	1																									1
Pope, Harrison																	1					1				2
Quesada, Jean-Louis																							1			1
Ranft, Karla																1										1
Reiber, Fabiola																					1					1
Robach, Paul																							1			1
Roustit, Matthieu																							1			1
Sagoe, Dominic																					1					1
Saugy, Martial																					1					1
Sayed, Khadiga																					1		4		1	6
Schaffer, Jay																	1									1
Schnuerch, Martin																					1					1
Schu, Kim																										1
Schröter, Hannes															1											1
Seifarth, Sebastian																		1								1
Simon, Perikles					1				1			1			2	1	1		1			1				9
Smith, Adam																		1								1

Stahlberger, Marius										1																1
Stamm, Hanspeter										1																1
Striegel, Heiko					1				1		1	1			1											5
Stubbe, Janin													1													1
Studzinski, Beatrix															1											1
Tareq, Aeshah																						1				1
Trebes, Gilles																							1			1
Ulrich, Rolf					1				1			1			1		1		1		1	1				8
Van der Heijden, Peter													1								1		4		1	7
Watkins, Stephen															1											1
Whitaker, Lisa															1											1
Wijekoon, Naveen																			1							1
Woertman, Liesbeth																				1						1
Zwingenberger, Stefan																		1								1
